# A common theme in extracellular fluids of beetles: extracellular superoxide dismutases crucial for balancing ROS in response to microbial challenge

**DOI:** 10.1038/srep24082

**Published:** 2016-04-12

**Authors:** René R. Gretscher, Priska E. Streicher, Anja S. Strauß, Natalie Wielsch, Magdalena Stock, Ding Wang, Wilhelm Boland, Antje Burse

**Affiliations:** 1 Department of Bioorganic Chemistry, Max-Planck-Institute for Chemical Ecology, Hans-Knoell-Str. 8, D-07745 Jena, Germany; 2 Research Group Mass Spectrometry/Proteomics, Max Planck Institute for Chemical Ecology, Jena, Germany

## Abstract

Extracellular Cu/Zn superoxide dismutases (SODs) are critical for balancing the level of reactive oxygen species in the extracellular matrix of eukaryotes. In the present study we have detected constitutive SOD activity in the haemolymph and defensive secretions of different leaf beetle species. Exemplarily, we have chosen the mustard leaf beetle, *Phaedon cochleariae*, as representative model organism to investigate the role of extracellular SODs in antimicrobial defence. Qualitative and quantitative proteome analyses resulted in the identification of two extracellular Cu/Zn SODs in the haemolymph and one in the defensive secretions of juvenile *P. cochleariae*. Furthermore, quantitative expression studies indicated fat body tissue and defensive glands as the main synthesis sites of these SODs. Silencing of the two SODs revealed one of them, *Pc*SOD3.1, as the only relevant enzyme facilitating SOD activity in haemolymph and defensive secretions *in vivo*. Upon challenge with the entomopathogenic fungus, *Metarhizium anisopliae*, *Pc*SOD3.1-deficient larvae exhibited a significantly higher mortality compared to other SOD-silenced groups. Hence, our results serve as a basis for further research on SOD regulated host-pathogen interactions. In defensive secretions *Pc*SOD3.1-silencing affected neither deterrent production nor activity against fungal growth. Instead, we propose another antifungal mechanism based on MRJP/yellow proteins in the defensive exudates.

Using oxygen in aerobic metabolism provides more energy per glucose than glycolysis does and hence constitutes an evolutionary advantage. Anyhow, molecular oxygen can be reduced to highly reactive by-products, termed reactive oxygen species (ROS), that can inflict damage on almost all molecules^1^. Oxygen is therefore a stringent selection criterion acting on organisms to sustain its reactive variants^2^,^3^,^4^. O_2_•^−^ is considered the “primary” ROS, which can further interact with other molecules to generate “secondary” ROS including hydroxyl radicals (•OH), hydroperoxyl radicals (HOO•) or hydrogen peroxide (H_2_O_2_)^5^,^6^. While organisms evolved antioxidant activity to counteract the deleterious effects (termed oxidative stress) of these oxygen variants, they have also developed mechanisms to use ROS as vital components in cellular signalling pathways or immune response^5^,^7^,^8^.

Superoxide dismutases (SOD, E.C. 1.15.1.1), common to all kingdoms of life, serve as first-line antioxidant enzymes^6^,^9^. They dismutate O_2_•^−^ into O_2_ and H_2_O_2_ with diffusion-limited rate constants. SODs are classified according to their metal co-factors and localization^10^. In eukaryotes two intracellular forms are known. The dimeric Cu/Zn-binding SODs (SOD1; 32 kDa) are localized in the cytoplasm, outer mitochondrial space and nucleus^11^, whereas the tetrameric MnSODs (SOD2; 89 kDa) is exclusively present in the inner mitochondrial space^12^. The most recently discovered members of the SOD family are the tetrameric Cu/Zn SODs (SOD3; EC-SOD; 135 kDa)^13^,^14^,^15^,^16^ functioning in the extracellular matrix.

Although SOD3 has been conserved among metazoans, the first functional evidence for a SOD3 in insects has been reported from the ant *Lasius niger* not before the 21st century^17^. And still, the understanding of the function of extracellular SOD isoforms in insects is incomplete and only a few examples have been published to date. For *Drosophila melanogaster* a SOD3 was reported to exhibit a protective effect against oxidative stress caused by ROS generating agents such as paraquat^18^, UV-radiation, and has an impact on life-span regulation^19^. Further, it has been assumed that extracellular SODs play a role in insect-parasitoid interactions^20^. Parasitoid wasps, for example, produce extracellular SODs and secrete them into their venom^21^. These exogenous SODs are suggested to increase the survival of the parasitoid eggs upon injection of the venom into an insect host during oviposition.

However, extracellular variants of SODs do not seem to play only a role in wasp venoms but can be found also in other insect defensive exudates. In the present study, we have identified a SOD3 isoenzyme in the proteome of the larval defensive secretions collected from the mustard leaf beetle *Phaedon cochleariae* (family Chrysomelidae, subtribe Chrysomelina). Within the Chrysomelina, the larvae of all species defend themselves by semiochemicals produced and released from specialized exocrine glands upon disturbance^22^,^23^,^24^,^25^. The defensive glands function as “bioreactors” where pre-toxins are enzymatically converted into biological active compounds. In *P. cochleariae*, the defensive iridoid chrysomelidial is converted from imported 8-hydroxygeraniol glucoside^26^,^27^,^28^,^29^,^30^,^31^. This transformation involves the hydrolysis of the glucoside and oxidation of the two primary hydroxy groups to produce the dialdehyde 8-oxogeranial, followed by a cyclisation step^29^,^32^,^33^,^34^,^35^. SODs could function as superoxide scavengers during the FAD dependent oxidation of 8-hydroxygeraniol or as a general protector of secretions’ components from oxidative stress during storage. Besides in *P. cochleariae*, oxidation reactions in the defensive secretions of larvae can also be found in related Chrysomelina species^29^,^36^,^37^,^38^.

By means of *P. cochleariae* larvae, we report the identification of a glandular extracellular SOD together with an additional extracellular isoform in the haemolymph. Given the open circulation in insects, the SODs are in contact with all tissues and, therefore, it is hypothesized that these enzymes have an overall function in the insects, for example, in the innate immunity. Their importance in ROS balancing mechanisms prompted us to investigate extracellular SODs and their role in opposing microbial invaders in the haemolymph as well as their possible function in the defensive larval exudates. As we have found constitutive extracellular SOD activity in secretions and haemolymph in related leaf beetle species we propose a widespread function of SOD3 in extracellular fluids of insects.

## Results

### Distribution of extracellular SOD activity among juvenile leaf beetles

**Figure 1:**
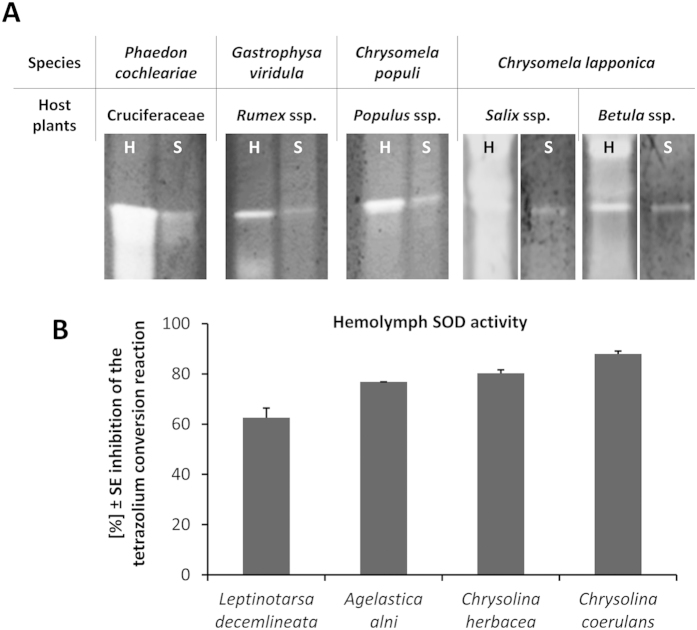
Detection of SOD activity in defensive exudates and haemolymph collected from larvae of different leaf beetle species. (**A**) From each species 1 μl haemolymph (H, left) and 0.3 μl secretion (S, right) was separated on native PAGE and functionally stained for SOD activity by using a qualitative zymography method based on the conversion of the tetrazolium salt, MTT. SOD activity inhibits the formation of a dark blue formazane dye and corresponds to white bands on a dark background. Here we show cropped gel sections. Full-length gels are presented in Supplementary Figs S14 and S15. (**B**) From pooled individuals of each species 0.8 μl of haemolymph was used in a 20 μl reaction mixture for the SOD assays using a quantitative microtiter plate method. SOD activity was determined colorimetrically at 440 nm as the inhibition of the reduction of the tetrazolium salt, WST-1 (n = 3).

In order to examine SOD activity in extracellular fluids of leaf beetles, we have carried out in gel activity assays. We detected activity in the defensive secretions as well as in the haemolymph of all analysed larvae which were collected from different related species of the subtribe Chrysomelina (Fig. 1A). Interestingly, the extracellular SOD activity in the larval secretions is independent from their pathway of deterrent production. The biosynthesis of deterrents usually involves an oxidation reaction, as in the case of *P. cochleariae*, *Gastrophysa viridula*, *Chrysomela populi*, willow-feeding *Chrysomela lapponica.* However, birch-feeding *C. lapponica* larvae are devoid of an oxidation step in the deterrent synthesis and could therefore surrender SOD activity in the secretions^38^,^39^,^40^. Further analyses of other larvae of leaf beetles including members of the genera *Chrysolina* (Chrysomelini, Chrysolinina) *Leptinotarsa* (Chrysomelini, Doryphorina) and *Agelastica* (Galerucinae, Sermylini) showed activity in their haemolymph (Fig. 1B). It is, therefore, reasonable to assume that extracellular SOD activity is a widespread phenomenon in leaf beetles, which did not emerge in just a single subtribe. For our further experiments, we have chosen the mustard leaf beetle, *P. cochleariae*, as case study to investigate the function of extracellular SODs in insects.

### Identification of extracellular Cu/Zn SODs in juvenile *Phaedon cochleariae*

Larval secretions as well as cell-free haemolymph were at first separated by 1D-SDS-PAGE. After recovering, the protein bands were then analysed by LC-MS^E^. For each of the two samples, the peptides derived from proteins of a molecular weight of 15 to 25 kDa were matched to data base proteins with similarity to Cu/Zn SODs (Sod_Cu (PF00080) EC 1.15.1.1). The full-length amplification and sequencing of the corresponding transcripts led to coding sequences of 516 bp for *Pc*SOD3.1 and 534 bp for *Pc*SOD3.2 for *P. cochleariae*. While *Pc*SOD3.1 has been identified by LC-MS^E^ analyses in the defensive secretions and haemolymph, *Pc*SOD3.2 has been determined only in the haemolymph (Supplementary Table S1).

The deduced amino acid sequences *Pc*SOD3.1 and *Pc*SOD3.2 possess about 50% identity with each other. For *Pc*SOD3.1 and *Pc*SOD3.2, N-terminal signal peptides were specified by cleavage site predictions suggesting those to be extracellular proteins. Further analyses of their structure by homology modelling using Phyre2^41^ revealed for both proteins active sites and binding motifs for the metal cofactors which are summarized in Table 1 and visualized in Supplementary Figs S1 and S2. *Pc*SOD3.2 possesses amino acid exchanges in position H85 and H102 into proline and asparagine, respectively.

In order to demonstrate the *in vitro* activity of *Pc*SOD3.1 and *Pc*SOD3.2, both enzymes have been expressed heterologously in *E. coli* and purified subsequently. In the enzyme assays, recombinant *Pc*SOD3.1 displayed an activity of 36% ± 8% whereas *Pc*SOD3.2 showed an activity of only 1% ± 0.6% (see Supplementary Fig. S3). Hence, *Pc*SOD3.2 seems to have a strongly reduced activity compared to *Pc*SOD3.1, most likely due to the amino acid exchanges in its active centre.

Phylogenetic analyses including intra- and extracellular Cu/Zn SODs encoding sequences inferred from our transcriptome reference libraries, other insect and mammalian homologs showed a clustering of the identified *Pc*Cu/Zn SODs into the group of extracellular SODs (Supplementary Fig. S4A). While one group of intracellular SODs cluster together, the other group is formed by the extracellular variants. Human SOD3 is grouped to the extracellular enzymes. Sequences similar to *Pc*SOD3.1 and *Pc*SOD3.2 were found in related Chrysomelina species with bootstrap values of 43% and 100%, respectively. Chrysomeline *Pc*SOD3.2 homologs also possess amino acid changes in the positions as in *Pc*SOD3.2 (Supplementary Fig. S4B). Remarkably, within the extracellular proteins conserved sequences were found with a high bootstrap value of 70%. A physiological function, however, has not been described as yet for any of these proteins.

### Localization of Cu/Zn SODs in juvenile *P. cochleariae*

We performed qPCR to analyse the transcript abundance of the extracellular SODs in *P. cochleariae* larvae. The defensive glands predominantly expressed *PcSOD3.1*. In the fat-body tissue, we detected a high expression of both *PcSOD3.1* and *PcSOD3.2* (Supplementary Fig. S5). Due to the relatively low mRNA levels in all the other tested tissues, including gut, Malpighian tubules, haemolymph containing haemocytes, and head we consider the defensive glands and fat body tissue as the main synthesis sites of *Pc*SOD3.1 and *Pc*SOD3.2.

In human SOD3, the C-terminus is highly rich in positively charged lysine and arginine residues that are involved in anchoring to heparan sulfate proteoglycans on cell surfaces and connective tissue matrix^42^,^43^,^44^. This C-terminal domain can be proteolytically cleaved to facilitate entrance to extracellular fluids. The two extracellular insect *Pc*SODs, however, lack such a heparin binding peptide (Supplementary Fig. S1). In order to test the membrane association of extracellular SODs, the tissues of Malpighian tubules and fat body from juvenile *P. cochleariae* have been separated into membrane and cytosolic fractions. All fractions exhibited SOD activity (Supplementary Fig. S6). By using LC-MS^E^ proteome analyses, we were, however, not able to detect extracellular SODs in any of the samples. Instead, we identified an additional Cu/Zn SOD (Supplementary Table S1). The corresponding full-length sequence does not have a predicted peptide signal suggesting that it is a cytoplasmic variant. It has been therefore named *Pc*SOD1 (Table 1). This *Pc*SOD1 is expressed in all tested larval tissues (Supplementary Fig. S5). The activity of recombinant *Pc*SOD1 has also been assayed *in vitro* and was found to be active with 59% ± 6% (Supplementary Fig. S3). As SOD1 has been reported to play an important role for ROS detoxification and immune response in other insects we have included *Pc*SOD1 into our further analyses as a positive control of RNAi effects^45^,^46^,^47^,^48^,^49^.

### Effects of *PcSOD3.1* silencing on the defensive secretions of *P. cochleariae*

**Figure 2:**
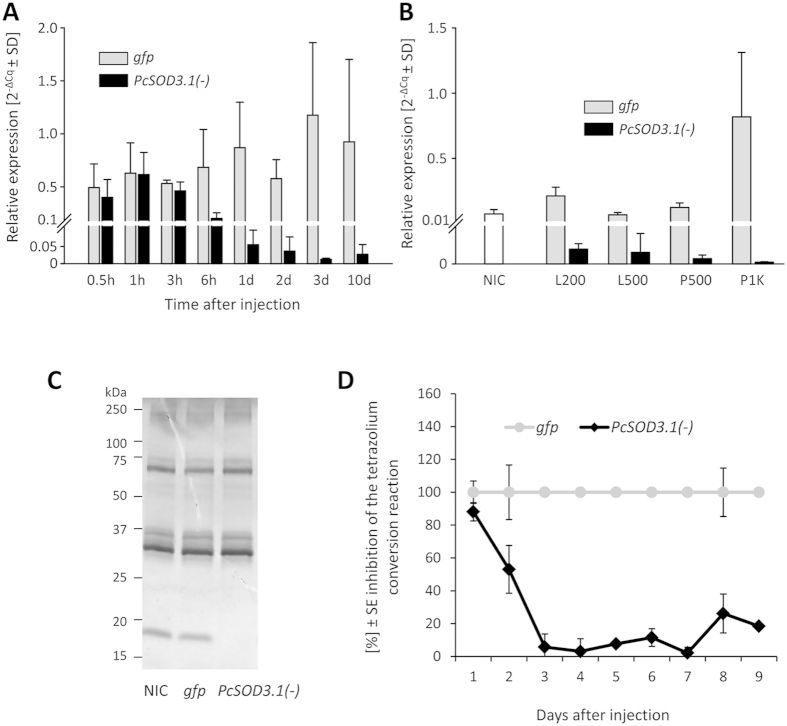
RNAi effects on transcript and protein abundance, and SOD activity in *P. cochleariae*. (**A**) Transcript abundance of *PcSOD3.1* after injection with 200 ng *PcSOD3.1*-dsRNA or *gfp* as a control, respectively. RNA from whole larvae has been used as template. The efficiency corrected Cq values of *PcSOD3.1*-silenced samples have been normalized to the reference genes *PcRPL6* and *PcRPS3* (n = 3). (**B**) Resulting transcript abundance of whole female adult beetles eight weeks after maturation, which were injected with *PcSOD3.1*-dsRNA or *gfp*-dsRNA during different developmental stages (n = 5). L200, larvae injected with 200 ng dsRNA; L500, larvae injected with 500 ng dsRNA; P500, pupae injected with 500 ng dsRNA; P1K, pupae injected with 1 μg dsRNA. (**C**) Coomassie stained SDS-PAGE with 0.3 μl of larval defence secretions from each experimental group, nine days after RNAi induction as described in (**A**). Here we show a cropped gel section. The full-length gel is presented in Supplementary Fig. S16. NIC, Non-injected control. (**D**) Time series of SOD activity of defence secretions, quantitatively determined by using a colorimetric microtiter plate assay. From pooled individuals from each experimental group 0.3 μl of defensive secretions was used in a 20 μl reaction mixture for the SOD assays. Values of the *PcSOD3.1*(−)-group were normalized to *gfp*-control (n = 3).

To study the function of extracellular SODs in leaf beetles *in vivo* we have tested RNAi by targeting the first candidate *PcSOD3.1*. We injected dsRNA*-PcSOD3.1* into each of early second-instar larvae. A significant reduction of the transcript in the whole larvae compared to the control group has been measured already 6 h after injection (29.5% ± 7.9), which continued dropping down to 1% ± 1 within 24 h and revealed to be stable until pupation 10 days later (0.6% ± 0.6) (Fig. 2A). Though the reduction of the transcript was stable even in adult beetles until their death (Fig. 2B), eight weeks after maturation (4.5% ± 7.4), the silencing effects were not transmitted into the F1-generation (Supplementary Fig. S7) (cf. Bucher *et al.*^50^).

Consistently with the reduction of the transcript level, both, the protein and the enzymatic activity, decreased rapidly in *P. cochleariae* larvae (Fig. 2C,D). *Pc*SOD3.1 was not detectable anymore in the defence secretome nine days after dsRNA*-PcSOD3.1* injection whereas the protein pattern of the remaining proteins compared to the controls remained unchanged. SOD activity of the secretion was reduced to 5.7% ± 7.1 compared to *gfp*-control and stayed reduced by more than 80% until prepupal stage.

In addition, we checked the amount of chrysomelidial in the defence secretion, as an indicator of an RNAi-related alteration of the *de novo* synthesis of this defence allomone, but observed no significant differences among the populations (Supplementary Fig. S8). Hence, the silencing of *PcSOD3.1* in the defensive secretions did not affect the production of the defensive chemistry.

### Comparing the silencing effects of extracellular and cytoplasmic Cu/Zn SODs in the secretions and haemolymph of *P. cochleariae*

**Figure 3:**
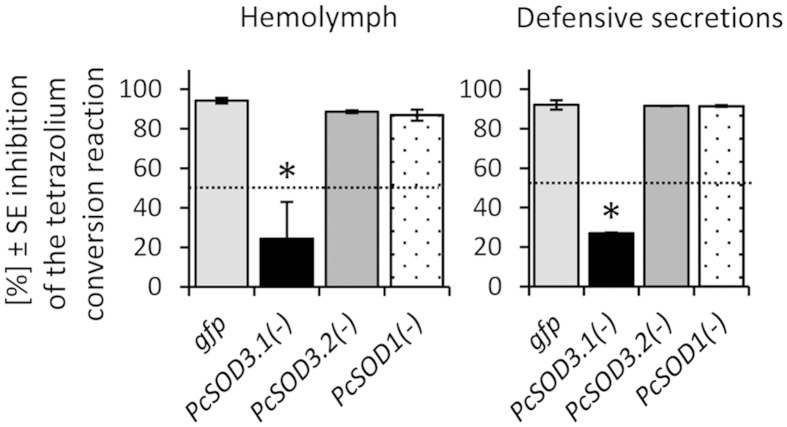
RNAi effects after individual silencing of Cu/Zn *PcSOD*s in extracellular fluids of *P. cochleariae* larvae. SOD activity was measured ten days after injection with 200 ng of *PcSOD3.1*-, *PcSOD3.2*-, *PcSOD1*- or *gfp*-dsRNA. The used colorimetric assay was based on the inhibition of the reduction of the tetrazolium salt, WST-1. From pooled individuals of each experimental group 0.5 μl of haemolymph (left graph) and 0.3 μl of defensive secretions (right graph) was assayed regarding SOD activity in a 20 μl reaction mixture (n = 3). Level of significance: *p < 0.05 (ANOVA on ranks, Turkey test).

Besides *PcSOD3.1* we have knocked-down *PcSOD3.2* and *PcSOD1* individually. By comparing the transcript levels of the *Cu/ZnPcSODs* we observed no significant co-silencing or up-regulation in whole larvae 10 days after dsRNA injection (Supplementary Fig. S9). A quantitative SOD assay revealed an activity significantly reduced only in the *Pc*SOD3.1(−)-group; *Pc*SOD3.1 seems to be the responsible enzyme for the constitutive SOD activity found in haemolymph and secretions (Fig. 3). Hence, no other SOD putatively existing in the genome seems to take over the function to the extent of *Pc*SOD3.1 in haemolymph and secretions after its silencing.

### Effect of Cu/Zn SOD silencing on beetle development and life-span

We induced RNAi in second-instar larvae and monitored long-term effects due to decreased SOD activity in *P. cochleariae*. The period of time in which individuals of one group remained in one developmental stage, appeared not to differ significantly in the transition from larval to pupal and to adult stage (Supplementary Fig. S10). Monitoring the longevity revealed that the silencing of *PcSOD1* resulted in an earlier death of individuals after 115 days compared to controls or the extracellular SOD-deficient groups (Supplementary Fig. S11). This observation is in accordance with papers showing an impact of SOD1 in life-span of e.g. *D. melanogaster* knockout mutants e.g.^49^. Statistic tests applied to our data, however, did not demonstrate significance for the observed phenomenon. Concluding, the probability of survival of *P. cochleariae* is not impaired by the silencing of the single *PcSOD* genes.

### Consequences of SOD-knock-down after microbial challenge

As O_2_•^−^ and hydrogen peroxide play important roles on innate immune response against potentially invasive organisms^45^,^51^, we tested whether the deficiency in one Cu/Zn *Pc*SOD alters survival of *P. cochleariae* larvae exposed to microbes after inoculation with the entomopathogenic fungus *M. anisopliae*, the Gram(−)bacterium *E. coli* K12 and the Gram(+)bacterium *M. luteus*.

**Figure 4:**
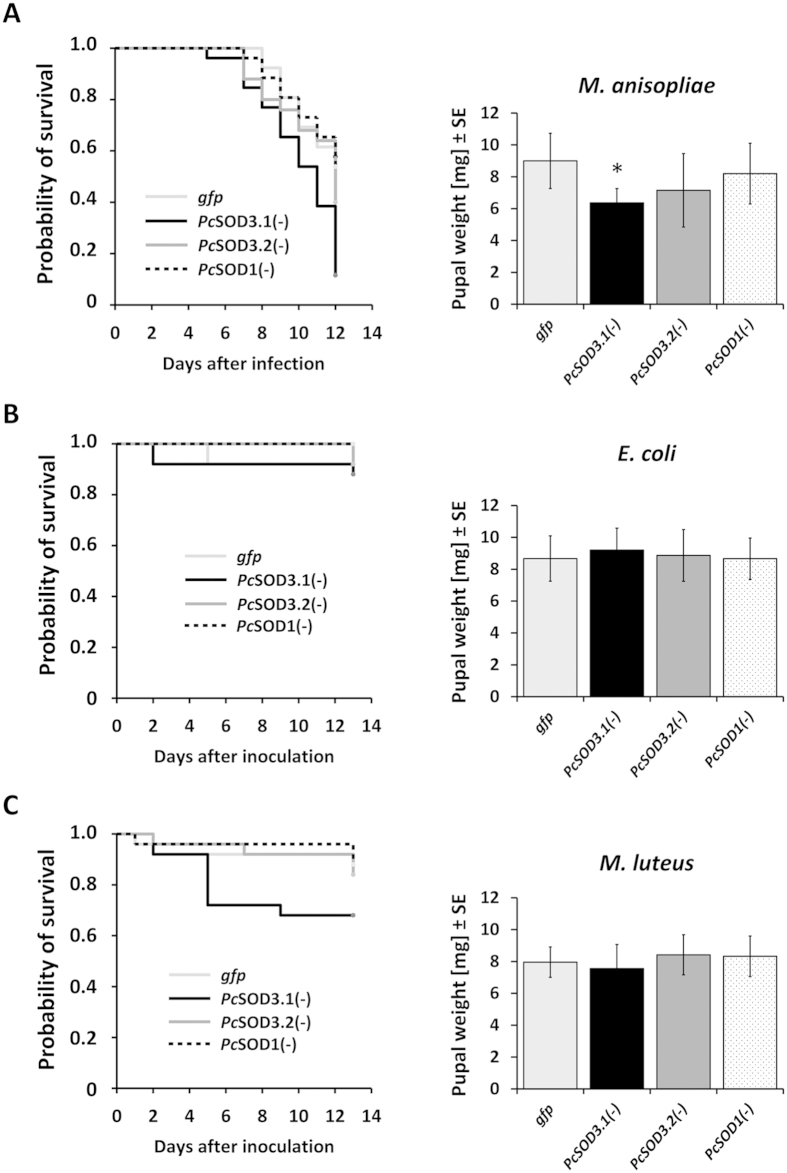
Immunologic consequences of Cu/Zn *Pc*SOD silencing in *P. cochleariae*. Thirty larvae were injected each with 200 ng of *PcSOD3.1*-, *PcSOD3.2-, PcSOD1-*dsRNA or *gfp*-dsRNA. Five days after this initial injection, larvae were challenged with microbes. The probability of survival is depicted as Kaplan-Meier curves by applying Gehan-Breslow statistic test. The weight of freshly emerged pupa was determined after RNAi silencing and infection of larvae. (**A**) Survival upon fungal challenge. After emptying of the defence reservoirs, the larvae of each group were submerged at the same time in the same solution of *M. anisopliae* 1*10^6^ conidia/ml. Overall level of significance: p = 0.014 (Gehan-Breslow test); p = 0.04 (Log Rank (Mantel-Cox)). Pairwise multiple comparison procedures for *gfp* vs. *Pc*SOD3.1(−): p = 0.04 (Holm-Sidak method). Pupal weight, level of significance: p = 0.023* (two-tailed t-test). (**B**) Survival after injection of 1*10^6^ *E. coli*-cells/200 nl. (**C**) Survival after injection of 1*10^6^ *M. luteus*-cells/200 nl.

Testing pathogenicity of *M. anisopliae*, we could observe a fast increase in mortality of all groups, starting 6 days after inoculation (Fig. 4A). The probability of the survival of the *PcSOD3.1*(−) group was significantly reduced compared to that of the other groups. Also the pupal weight was reduced to average 6 mg which was significantly less than the 9 mg of *gfp* control. Hence, the *PcSOD3.1*(−) larvae suffering during the whole larval phase from fungal infection were possibly compromised in optimal nutrition compared to each of the other groups with intact *Pc*SOD3.1 enzyme.

LC-MS^E^ analyses of haemolymph revealed the induction of antimicrobial peptides as part of the humoral defence upon microbial challenge (Supplementary Table S2)^52^,^53^,^54^,^55^. For example, a protein sequence with a similarity of 77% to defensin from *Sitophilus zeamais*^56^ was detectable only in the infected haemolymph samples and not in the control. A protein, 61% similar to *S. zeamais* attacin C^56^, was about 20-fold increased after *M. anisopliae* infection. And also an acaloleptin A similar protein (83% similarity to the protein from *Acalolepta luxuriosa*^57^) was about 7-fold induced compared to the non-infected control. However, the antimicrobial peptides could apparently not prevent a lethal phenotype upon fungal infection.

Further time-dependent expression analyses comparing fungal-infected and non-infected larvae deficient in Cu/Zn SOD activity showed significant induction of *PcSOD3.2* and *PcSOD1* expression two days after inoculation (Supplementary Fig. S12). *PcSOD3.1* was not significantly induced over a period of six days. LC-MS^E^ analyses of the haemolymph of *PcSOD3.1*(−) larvae demonstrated that there is no induction of any other extracellular SOD upon infection compared to non-infected *PcSOD3.1*(−) larvae (Supplementary Table S3).

Because RNAi is systemic in beetles, a loss of *Pc*SOD3.1 in the defensive secretions could have an effect on the germination of the conidia on the surface of the larvae. Agar diffusion tests showed, however, that silencing of *PcSOD3.1* did not alter antifungal activity of the defensive secretions (Supplementary Fig. S13A). It is therefore reasonable to assume, that constitutive *Pc*SOD3.1 plays a pivotal role for ROS homeostasis in the haemolymph in immune response to fungal challenge. By using LC-MS^E^ analyses we have identified in the larval defensive exudates a protein similar to members of the yellow-like protein family characterized by the presence of a major royal jelly domain^58^,^59^ (Supplementary Table S4). In pilot experiments its silencing caused the loss of the antifungal activity of larval secretions against *M. anisopliae* (Supplementary Fig. S13B). Hence, the mode of action of antifungal activity in defensive secretions differs from that found in the haemolymph and is currently under investigation.

In case of inoculation by 1*10^6^ *E. coli* cells, we could not detect any difference on the survival or the pupal weight between the groups (Fig. 4B). In a similar experiment, using 1*10^6^ of *M. luteus* cells, we could detect a reduced, but statistically not significant, survival in the *PcSOD3.1*(−) group compared to *gfp* and *PcSOD1*(−) and *PcSOD3.2*(−) groups (Fig. 4C).

LC-MS^E^ analyses of haemolymph revealed also the induction of antimicrobial peptides after *E. coli* and after *M. luteus* infection, including defensin, attacin C (50-fold induced compared to the non-infected control), and also an acaloleptin A similar protein (4–10-fold induced compared to the non-infected control) (Supplementary Table S2). Apparently, in our experiments the lack of SOD activity did not interfere significantly with the antibacterial activity of the immune response in the insects most likely due to the action of the antimicrobial peptides.

## Discussion

The insect’s innate immune system is comprised of both cellular and humoral elements^60^,^61^,^62^,^63^,^64^. Invasive organisms that enter the host’s hemocoel encounter reactive haemocytes and an array of specific, e.g. antimicrobial peptides^52^,^54^,^65^,^66^, and non-specific, e.g. ROS, cytotoxic molecules^45^,^51^. Particularly, SODs have a paramount role in balancing ROS in an organism. We have identified two extracellular SODs, *Pc*SOD3.1 and *Pc*SOD3.2, and one cytosolic variant, *Pc*SOD1, in juvenile *P. cochleariae*. Unlike the human extracellular SODs, the extracellular isoforms from the beetles are most likely not attached to the extracellular matrix, but secreted as soon as they are synthesized. This phenomenon has already been described for the extracellular proteins of *Drosophila*^19^. Fat body and defensive glands of juvenile *P. cochleariae* are suggested to be the main production sites of the two extracellular SODs. On the basis of *in vitro* activity assays, *Pc*SOD3.1 displayed a higher activity compared to the activity of *Pc*SOD3.2. As *Pc*SOD3.2 is present in the haemolymph in much higher quantity compared to *Pc*SOD3.1, we hypothesize a function for *Pc*SOD3.2 in regulating ROS equilibrium in the haemolymph by e.g. trapping H_2_O_2_.

Processes causing ROS production in insects include the immune responses against microbes^67^,^68^. Our data show the vital role of *Pc*SOD3.1 in the haemolymph particularly against entomopathogenic fungi compared to treatments with bacteria. Immune response against fungal infection includes melanisation leading to the physical encapsulation of intruders in a dense melanin coat which is accompanied by the production of ROS together with the redoxactive melanogenic intermediates^63^,^69^,^70^,^71^. In our opinion, a direct participation of *Pc*SOD3.1 in the phenoloxidase cascade leading to melanin capsules could be excluded by the fact, that O_2_•^−^ is needed to drive melanisation. It has been shown, that the enzymatic oxidation of DOPA as intermediate of the melanisation cascade is SOD sensitive *in vitro*^72^, and a SOD in the process of encapsulation is contra-productive since it slows down melanisation^73^. Instead, we hypothesize two possible functions for *Pc*SOD3.1 identified in the beetle’s haemolymph. First, it could be intended to produce H_2_O_2_ which will fuel Fenton reactions. Second, H_2_O_2_ serves as signal intermediate, necessary for the transcriptional regulation of an adequate immune response^74^. H_2_O_2_ signalling, which is involved in immune response, proceeds via Nox-enzymes. Superoxide anions have to be converted in the extracellular space to H_2_O_2_ to be active in the cytosol after reimport^74^. This path is often described to rely on spontaneous dismutation of O_2_•^−^ but SODs work an order of magnitude faster yielding H_2_O_2_ and prohibit the depolarization of the membrane due to too many negative charges^75^,^76^. To conclude, if *Pc*SOD3.1 is missing in the haemolymph upon fungal infection, the melanisation cascade may be activated anyway, but the production of antimicrobial radicals or H_2_O_2_ for the regulation of downstream reactions leading to an effective immune response might be unbalanced.

Due to their sugar content, the larval defensive secretions of *P. cochleariae* could be a culture medium for microbes^34^. Therefore, it is not surprising that the secretions contain components of antimicrobial activity. For example, it has been demonstrated that chrysomelidial possesses an effect against Gram(−)bacteria and yeast^77^. In agar diffusion tests using defensive secretions from *Pc*Yellow-like(−) larvae against the entomopathogen *M. anisopliae* we found antifungal activity mediated presumably by a yellow-like protein. To date, the function of these proteins in insects is, however, largely not understood^59^. In *D. melanogaster*, for example, yellow members seem to be involved in the process of melanization^78^. In *Apis mellifera* these proteins are found as a major component in royal jelly, a substance which is fed to larvae for triggering their development into queens^58^. Yellow proteins have also been identified in the bee venoms, but a function has not been described as yet^79^. To what extent these proteins in bee venoms also have antifungal effects analogous to the defensive secretions, needs to be tested in the future.

In the chrysomelidial pathway, most likely *PcS*OD3.1 could have assisted the GMC oxidoreductase, *Pc*8HGO, being active in the defensive secretions^32^. But on the one hand, it has been shown, that the GMC oxidoreductases are producing directly H_2_O_2_ as a by-product of oxidation, without superoxide intermediates^80^. On the other hand, as the silencing of *PcSOD3.1* did not interfere with the amount of chrysomelidial produced, we are not able to infer a direct function of *PcS*OD3.1 for deterrent production from our experiments. Regardless, a possible function of *Pc*SOD3.1 could be the general protection of other secretions’ components, not yet identified, from oxidative stress during storage. This may explain why SOD activity is present in the defensive exudates of different chrysomeline species independent from the pathway of deterrent synthesis.

In summary, our results demonstrate that insects secrete extracellular SODs constitutively into extracellular fluids and that these proteins contribute to fungal defence in their innate immunity. As extracellular SOD activity seems to be a widespread phenomenon in the open circulation of insects, we created a suitable source on which to base current and future concepts regarding SOD regulated antimicrobial response in ecologically relevant non-model organisms.

## Methods

Information on biological replicates and the individuals pooled within is given in the figure legends. Statistical calculations are indicated in the figure legends; Excel (Microsoft, Redmond, WA, USA), SPSS (IBM, Armonk, NJ, USA) and SigmaPlot (Systat Software, Inc., CA, USA) have been used for the calculations. All primer used in this study are listed in Supplementary Table S5. The accession numbers of all sequences identified from *P. cochleariae* and all sequences used in this study are listed in Supplementary Table S6. All chemicals are purchased from Serva (Heidelberg, Germany), Sigma-Aldrich (St. Louis, MO, USA) or Carl Roth (Karlsruhe, Germany), if not stated other.

### Beetle rearing and sample collection of extracellular fluids

*P. cochleariae* was reared on *Brassica rapa* subsp. *pekinensis* “Cantonner Witkrop” (Quedlinburger Saatgut, Quedlinburg, Germany) in a Snijder chamber (Snijders scientific, Tilburg, Netherlands) in a light/dark cycle of 16 h light and 8 h darkness (LD 16/8) and 13 °C/11 °C ± 1 °C, 70% humidity. After microbial infection and during developmental experiments the insects were incubated at LD 14/10, 21 ± 2 °C, 70% humidity.

Larval secretions were collected in glass capillaries (i.d.: 0.28 mm, o.d.: 0.78 mm, length 100 mm; Hirschmann, Eberstadt, Germany). Secretions were weighed in the sealed capillaries on an ultra-microbalance (Mettler-Toledo, Greifensee, Switzerland) three times; the weight of the capillaries was subtracted and the final weight was averaged. Sealed capillaries were stored at −20 °C until needed. Haemolymph from *P. cochleariae* as well as from juveniles from related leaf beetle species was collected in graduated 5 μl glass capillaries (Hirschmann, Eberstadt, Germany) after scissoring of one meta-thoracal legs at the coxa and also stored at −20 °C after sealing.

### Identification of SOD encoding sequences and their phylogenetic relationships

For the identification of putative Cu/Zn SODs we analysed computationally the transcriptome libraries of the three Chrysomelina species *P. cochlearia*e^81^, *C. populi*^82^, and *C. lapponica*^32^. Using tool “transeq” from package EMBOSS (V6.3.1), the cDNAs of these libraries were translated into all six possible open reading frames. We annotated these protein sequences by applying pfamscan against the protein families’ database (Pfam, updated in Jan. 2013) with default parameters^83^. Those sequences that were annotated to possess a Sod_Cu domain (PF00080) and to be longer than 130 amino acids were selected for further analyses. Sequences were also searched via BLAST approach against the NCBI non-redundant database to validate the prediction^84^. In addition, SignalP (SignalP 4.1: http://www.cbs.dtu.dk/services/SignalP/)^85^ has been used to identify signal peptides. The tool GlycoEP has been used to predict N-Glycosylation sites (http://www.imtech.res.in/raghava/glycoep/)^86^. Selected sequences have been further analysed by using Phyre2 (http://www.sbg.bio.ic.ac.uk/phyre2/html/page.cgi?id=index)^41^.

To analyse the phylogenetic relationships of the identified Cu/Zn SOD sequences from the three Chrysomelina leaf beetle species and other insects, a phylogenetic tree has been constructed. In addition to the Chrysomelina sequences mentioned above, we included predicted Cu/Zn SODs of *G. viridula* and *Leptinotarsa decemlineata* identified in corresponding transcriptome libraries^87^, and of other Coleoptera, Hemiptera, Diptera, Hymenoptera, Lepidoptera, and Mammalia. The sequence of a bacterium was used as outgroup. The accession numbers of these sequences are listed in Supplementary Table S6. The identified protein sequences were aligned by using MAFFT (v7.023b) with default parameters and L-INS-i method. Afterwards, the randomized accelerated maximum likelihood tool (RAxML, V7.2.8) was applied with the parameter of ARTREVF (bootstrap value = 1000) to generate the phylogenetic tree.

### Quantitative real time PCR

For generation of cDNA insects were dissected in 0.9% NaCl solution under an stereomicroscope (Zeiss, Jena, Germany), where fat body, gut and Malpighian tubules were frozen directly on the wall of liquid nitrogen cooled reaction tubes. Defensive glands were collected with a pipette in 200 μl RNAlater (Qiagen, Hilden, Germany). Whole larvae and adults were placed alive in 2 ml safe-lock reaction tubes (Eppendorf) containing 6–10 silica beads (Ø2mm) and poured with 200 μl lysis-buffer (kit see below). These samples were homogenized using a genogrinder2010 (Spex samplePrep, Metuchen, NJ, USA). All samples were stored until further processing at −80 °C. RNA extraction and cDNA synthesis were performed according to^88^ using the RNAqueous kit (Ambion, Thermo scientific), superscript III enzyme and oligo dT_(12–18)_ primer (both Invitrogen, Thermo scientific). Quality was assessed by TA gel electrophoresis, and the A_260_/A_280_ value.

cDNA from haemolymph, head, gut, Malpighian tubules, fat body and accessory defence glands, whole larvae or adults of *P. cochleariae* was used as a template for expression analysis of *Cu/Zn PcSOD*s using a CFX96 qPCR system (Biorad, Hercules, CA, USA) and Brilliant III SYBR green Mastermix (Agilent, Santa Clara, CA, USA). Cq values of *PcSOD*s from three biological and two technical replicates were normalized to *PcRPL6* and *PcRPS3* (Supplementary Tables S5 and S6). Resulting data were analysed using qBASE PLUS software^89^. Assays were performed following the MIQE guidelines^90^.

### Cloning of Cu/Zn *Pc*SODs

Coding sequences of *PcSOD3.1*, *PcSOD3.2*, *PcSOD1*, and *PcYellow-like* were amplified from a cDNA pool of all tissues using *Pf*x-Polymerase (Invitrogen, Thermo scientific). The fragments were cloned into pIB/V5-HIS TOPO vectors (Invitrogen, Thermo scientific), which is lacking T7-promotor sites, interfering with dsRNA synthesis. For that, this part of each coding region has been chosen, which resulted to be unique after off-target-prediction^88^. These fragments were amplified using PCR with primers containing both a 5′-T7-promotor-sequence. Also, the unique part of the coding sequence of the green fluorescent protein from pcDNA3.1/CT-GFP-TOPO (Invitrogen, Thermo scientific) was subjected to the described protocol and cloned into a pIB-vector. The cloning products were primarily amplified using *E. coli* TOP10F’ cells (Invitrogen, Thermo scientific) and sequenced, to confirm unaltered dsRNA templates.

### dsRNA synthesis

Sequenced pIB-plasmids were used to re-amplify DNA templates via PCR. The amplicons were subjected to T7-polymerase based *in vitro* transcription reactions (MEGAscript RNAi kit, Ambion, Thermo scientific) according to manufacturer’s instructions. The resulting dsRNA was eluted after nuclease digestion three times with 50 μl hot injection buffer (3.5 mM Tris-HCl, 1 mM NaCl, 50 nM Na_2_HPO_4_, 20 nM KH_2_PO_4_, 3 mM KCl, 0.3 mM EDTA, pH 7.0). For control of the influence of the activation of the RNAi-machinery, we by default raised a control group injected with corresponding amounts of dsRNA according to the *gfp* nucleotide sequence, which has no counterpart in *P. cochleariae* transcriptome. The concentration of dsRNA was measured spectrophotometrically, calculated with A_260_ = 1 = 45 mg/ml and adjusted to 2 μg/μl. The quality of dsRNA was checked by TBE-agarose-electrophoresis.

### RNAi induction

Second instars of *P. cochleariae* with three mm body length and >1.0 mg body weight were injected individually with 0.2 μg of dsRNA about five days after hatching. Injections were accomplished with ice-chilled larvae using a Nano2010 injector (WPI, Sarasota, FL, USA) driven by a three-axis micromanipulator mounted to a stereomicroscope (Zeiss). The larvae were injected dorso-medial between the pro- and mesothorax.

### Heterologous expression of Cu/Zn *Pc*SODs

For the *in vitro* functional validation of Cu/Zn *Pc*SODs, we used heterologously expressed 6xHIS-N-terminal tagged proteins. The coding sequences of *PcSOD3.1*, *PcSOD3.2*, and *PcSOD1* omitting the signal peptide-sequence in cases of the extracellular variants were cloned directionally into pET100/D TOPO vectors (Invitrogen, Thermo scientific). Using the Calbiochem Autoinduction system 1 (Merck, Darmstadt, Germany), recombinant (r)*Pc*SOD1, r*Pc*SOD3.1, and r*Pc*SOD3.2 were produced in *E. coli* strain BL21(DE3)star (Invitrogen, Thermo scientific) and purified according to Frick *et al.*^27^. A negative control has been included with a recycled pET100-TOPO vector with no insert.

### Microbial infection

L2-larvae of *P. cochleariae* were treated with *Cu/Zn PcSOD-*dsRNA or *gfp*. Five days later, developed third-instar larvae from were injected with bacteria or dipped into fungal conidia solution. Hereafter they were kept at room temperature, to allow the pathogens to develop.

*Metarhizium anisopliae* (DSM-1490, DSMZ, Braunschweig, Germany) was maintained on potato-dextrose-agar (Sigma, 2% glucose), and mycelia were used for subsequent propagation. Conidia were harvested from liquid culture (potato-dextrose, 200 ml in 500 ml Erlenmeyer flask), which was incubated at room temperature for three weeks without shaking, after overnight agitation of cultures with a 10 cm magnetic stirring bar at 4 °C. Mycelium was separated by the filtration of the suspension through fine gauze. The supernatant, containing the conidia was centrifuged (10.000 × g, 4 °C, 10 min.) and washed twice with 0.1% aqueous tween20 solution. The concentration of conidia was adjusted to 1*10^6^/ml (hemocytometer) and infection was performed according to Rostas *et al.*^91^. All larvae of one RNAi-induced group were submerged for 30 sec. in 1 ml of conidia suspension after swabbing the larvae on lab tissue, to get rid of defence secretions. The suspension was poured on filter paper and the larvae were air dried for 1 min. Only those larvae were assigned as killed by the fungus, which showed mycelia growth out of the carcass after incubating the corps on humid sterile filter paper in sealed petri dishes.

For bacterial infections, RNAi has been induced and 1*10^6^ cells of *E. coli* K12 (NEB, Ipswich, MA, USA) or *Micrococcus luteus* (DSM 20030, DSMZ, Germany) in 100 nl were injected into third-instar larvae. Both bacterial species were maintained on LB-agar-plates and fresh bacteria were purified from inoculated liquid LB cultures, agitating for four days at 37 °C, 200 rpm. The cells were pelleted (5 min, 8.000 × g, 4 °C) in 50 ml Falcon tubes and washed twice in Ringer solution^92^ and adjusted to 1*10^10^ cells/ml. The experiment ended again with the death or the successful pupation of the species.

Probabilities of survival after the treatments have been statistically analysed by using Kaplan-Meier estimation with Gehan-Breslow statistic test and pairwise multiple comparison procedures (Holm-Sidak method) by applying SigmaPlot software (version 11.0). Log Rank (Mantel Cox) tests have been carried out by using SPSS software (version 17.0).

Haemolymph samples for analysing antimicrobial peptides by LC-MS^E^ were collected from third-instar larvae four days after challenging with bacteria or *M. anisopliae* as described above.

Antifungal activity of secretions was testes in agar diffusion assays using conidia of *M. anisopliae*. Secretions of *PcSOD3.1* or *yellow-like* silenced larvae were tested eight days after the triggering of RNAi. Potato-dextrose-agar (Sigma, 2% glucose) was inoculated with 1*10^6^ conidia/100 ml agar.

### Sample preparation for protein analyses

Samples of snap-frozen tissues of 8–10 larvae were placed into 50 μl Ringer’s solution with Protease-Inhibitor Mix HP (Serva, Heidelberg, Germany). Tissues were grinded with sea sand. After the centrifugation of the samples (10 min., 2000 × g, 4 °C), supernatant was stored at −20 °C in a fresh reaction tube.

Haemolymph samples were also centrifuged and the supernatant checked for being cell-less with a transmitted light microscope before activity assays and proteome analyses. Protein concentrations were determined spectrophotometrically or by using bicinchoninic acid assay (Pierce, Thermo scientific). For membrane protein isolation the Native Membrane Protein Extraction Kit (Calbiochem, Proteo Extract) was used. Frozen tissue samples were processed in accordance with the manufacturer’s instructions.

### Protein digestion and nanoUPLC-MS/MS analysis

Protein samples were divided in two groups: for shotgun LC-MS/MS analysis and for Gel-LC-MS/MS approach. For gel-based LC-MS/MS analysis, protein samples were separated by any-kD gradient gels (Bio-Rad Laboratories, Munich, Germany) in SDS-PAGE^93^. Protein bands of Coomassie Brilliant blue R250 stained gels were cut from the gel matrix and tryptic digestion was carried out as described^94^. For LC-MS analysis the extracted tryptic peptides were reconstructed in 10 μl aqueous 1% formic acid. For semi-quantitative analyses of antimicrobial peptides, Waters MassPREP BSA digestion standard (Waters, Manchester, UK) as internal standard was added to the samples in the concentration of 50 fmol/μl.

For shotgun analysis the samples were subjected to TCA precipitation according to the protocol from Gorg *et al.*^95^. Prior precipitation each sample was spiked with BSA (Pierce, Thermo scientific) used as an internal standard for protein quantification, for 1 μg of protein 1 pmol of BSA was spiked. The protein pellets subjected for shotgun-LC-MS/MS were suspended in 100 mM NH_4_HCO_3_ by vortexing, heated to 90 °C for 20 min, cooled on ice and diluted with occasional vortexing with methanol to produce a sample solution containing 60% methanol. The samples were not fully dissolved and therefore stepwise digested with trypsin at a trypsin/protein ratio of 1:30 at 37 °C for approximately 12 hours.

The samples were acquired on a nanoAcquity UPLC system on-line connected to a Q-ToF Synapt HDMS mass spectrometer (Waters, Manchester, UK). The peptides were concentrated on a Symmetry C18 trap-column (20 × 0.18 mm, 5 μm particle size) using a mobile phase of 0.1% aqueous formic acid at a flow rate of 15 μL min^−1^ and separated on a nanoAcquity C18 column (200 mm × 75 μm ID, C18 BEH 130 material, 1.7 μm particle size) by in-line gradient elution at a flow rate of 0.350 μl min^−1^ using an increasing acetonitrile gradient from 1% to 95% B over 90 min (Buffers: A, 0.1% formic acid in water; B, 100% acetonitrile in 0.1% formic acid).

The eluted peptides were transferred into a Synapt HDMS Q-Tof tandem mass spectrometer equipped with a nanolockspray ion source (Waters, Manchester, UK). LC-MS data were collected using data-independent LC-MS^E^ analysis^96^. Full scan LC-MS data were collected using alternating mode of acquisition: low energy (MS) and elevated energy (MS^E^) mode over 1.5 sec intervals in the range m/z of 50–1700 with an interscan delay of 0.2 sec. A reference compound, human Glu-Fibrinopeptide B [650 fmol/mL in 0.1% formic acid/ACN (v/v, 1:1)], was infused through a reference sprayer at 30 s intervals for external calibration. The data acquisition was controlled by MassLynx v4.1 software (Waters).

### Data processing, protein identification and quantification

The acquired continuum LC-MS^E^ data were processed using ProteinLynx Global Server (PLGS) version 2.5.2 (Waters) to generate product ion spectra for database searching according to Ion Accounting algorithm described^97^. The thresholds for low/ high energy scan ions and peptide intensity were set at 150, 30 and 750 counts, respectively. The processed data were searched against the *P. cochleariae* protein subdatabases constructed from in-house transcriptome-database by their translation from all six reading frames combined with Swissprot database downloaded on 13 August, 2011 from http://www.uniprot.org/. The database searching was performed at a False Discovery Rate (FDR) of 4%, following searching parameters were applied for the minimum numbers of: product ion matches per peptide (3), product ion matches per protein (5), peptide matches (1), and maximum number of missed tryptic cleavage sites (1). Searches were restricted to tryptic peptides with a fixed carbamidomethyl modification for Cys residues. Proteins were quantified using the top 3 matched peptides (Hi3 method) from the spiked internal standard (BSA) as described by Silva *et al.*^98^

### SOD activity assays

SOD activity was determined by a qualitative in gel activity assay (in gel zymography) and a quanitative SOD microtiter plate assay. For conducting this zymography method, native gels were loaded with 15 μl of protein sample mixed with nondenaturing, nonreducing loading buffer. After performing electrophoresis at 150 V in a Native-PAGE running buffer, gels were incubated in the assay solution (80 ml 0.5 M Tris-HCl, 10 mg 3-[4,5-dimethylthiazol-2-yl]-2,5-diphenyltetrazolium bromide (MTT), 3 mg 5-methylphenazinium methyl sulfate (PMS), pH 8.0) for 15 min at 4 °C in the dark. Gels were then exposed to sunlight. SOD activity inhibited the reduction of the tetrazolium salt into the formazan dye and got visible, thus, as yellow bands on a dark-blue background.

Quantitative SOD activity assays, based on the conversion of the tetrazolium salt, WST-1 (2-(4-iodophenyl)-3-(4-nitrophenyl)-5-(2,4-disulfophenyl)-2H-tetrazolium, monosodium salt) into a formazan dye upon reduction with a superoxide anion, were carried out by using the SOD Determination Kit (Sigma-Aldrich, MO, USA) following the instructions of the manufacturer. The SOD standard inhibition curve was determined according to the manual with bovine erythrocyte SOD (15000 U/ml) and an incubation time of 20 min. Data sets were statistically evaluated by applying ANOVA on ranks (Kruskal-Wallis One Way Analysis of Variance on Ranks) together with a pairwise multiple comparison procedure (Tukey Test) by using SigmaPlot software (version 11.0).

## Additional Information

**How to cite this article**: Gretscher, R.R. *et al.* A common theme in extracellular fluids of beetles: extracellular superoxide dismutases crucial for balancing ROS in response to microbial challenge. *Sci. Rep.* **6**, 24082; doi: 10.1038/srep24082 (2016).

## Supplementary Material

Supplementary Information

## Figures and Tables

**Table 1:** Main characteristics of Cu/Zn SOD protein sequences identified in *P. cochleariae*.

	*Pc*SOD3.1	*Pc*SOD3.2	*PcS*OD1
Protein (aa)	171	177	153
Signal peptide	Yes (aa 1–16)	Yes (aa 1–20)	no
Molecular mass (kDa)	17.8	18.8	15.9
N-Glycosylation	N in position 36	N in position 43	N in position 23
Cysteine residues forming disulphide bonds	Cys74-Cys163	Cys79-Cys169	Cys56-Cys144
Zn^2+^ binding site	H80, H88, H97, D100	P85, H93, N102, D105	H62, H70, H79, D82
Cu^2+^ binding site	H63, H65, H80, H137	H68, H70, P85, H143	H45, H47, H62, H119
Active site	H63, H65, H80, H97, D100, H137	H68, H70, P85, N102, D105, H143	H45, H47, H62, H79, D82, H119
