# Supplementary material for: A common theme in extracellular fluids of beetles: extracellular superoxide dismutases crucial for balancing ROS in response to microbial challenge
Source: Sci Rep. 2016 Apr 12;6:24082. doi: 10.1038/srep24082 (PMC4828634; doi:10.1038/srep24082)
Supplement: Supplementary Information [file srep24082-s1.pdf]

Supplementary material: figures and tables

A common theme in extracellular fluids of beetles: extracellular superoxide dismutases crucial for balancing ROS in response to microbial challenge

René R. Gretscher[a], Priska E. Streicher[a], Anja S. Strauß[a], Natalie Wielsch[b], Magdalena Stock[a], Ding Wang[a], Wilhelm Boland[a] and Antje Burse[a*]

[a] Department of Bioorganic Chemistry, Max-Planck-Institute for Chemical Ecology, Hans-Knoell-Str. 8, D-07745 Jena, Germany

[b] Research Group Mass Spectrometry/Proteomics, Max Planck Institute for Chemical Ecology, Jena, Germany

*Corresponding author. Phone: ++49(0) 3641-571265, Fax: ++49(0) 3641-571202, E-mail: aburse@ice.mpg.de

SUPPLEMENTARY FIGURES

```
                                                      50
              ....|....|....|....|....|....|....|....|....|....|
PcSOD3.1      -----------------------------------MFKFAVLAALIT---
PcSOD3.2      -----------------------------------MFKFVVAVVLLSGTY
PcSOD1        --------------------------------------------------
HsSOD1        --------------------------------------------------
HsSOD3        MLALLCSCLLLAAGASDAWTGEDSAEPNSDSAEWIRDMYAKVTEIWQEVM

                                                     100
              ....|....|....|....|....|....|....|....|....|....|
PcSOD3.1      --ITYAQRSAVVYLFDPSG-----ASGVHGNLTFEQR--DSQIQISGEVH
PcSOD3.2      QFCNCDVHSATVRLYDPTG-----NTEVTGELNFTQV--ENGVRITGEIH
PcSOD1        ------MPTKAVCVLNG---------EVKGTVFFNQANSTSPVEVTGEIT
HsSOD1        ------MATKAVCVLKGDG-------PVQGIINFEQKESNGPVKVWGSIK
HsSOD3        QRRDDDGALHAACQVQPSATLDAAQPRVTGVVLFRQLAPRAKLDAFFALE

                                      *                      150
              ....|....|...*|*...|....|....|*...|...*|....|..*.|
PcSOD3.1      GLTPGKHG----FHVHQLGNIGLGCLGTGGHFNPHNKHHGAPTDKERHVG
PcSOD3.2      GIHEGKHG----GHVHVDADISKGCIGAGGPFNPHNVRHAGPDDEIRNTG
PcSOD1        GLQKGLHG----FHVHEFGDNTNGCISAGPHFNPRNVEHGGPDHDIRHIG
HsSOD1        GLTEGLHG----FHVHEFGDNTAGCTSAGPHFNPLSRKHGGPKDEERHVG
HsSOD3        GFPTEPNSSSRAIHVHQFGDLSQGCESTGPHYNPLAVPHP------QHPG

                                                             200
              *...|....|....|....|....|....|....|...*|....|....|
PcSOD3.1      DLGNIVADATGVAHVHIEDDVIAL-QGNHNIIGRAMVVHAGEDDLGRGGQ
PcSOD3.2      DLGNVVGDRFGVAHIDVVDKRIKLNKGIDGIIGRGFVIHEKEDDLGRGNN
PcSOD1        DLGNIQATDNGVAKVNIKDKFISL-EGENSIIGRTVVVHADPDDLGQGGH
HsSOD1        DLGNVTADKDGVADVSIEDSVISL-SGDHCIIGRTLVVHEKADDLGKGGN
HsSOD3        DFGNFAVRDG--SLWRYRAGLAASLAGPHSIVGRAVVVHAGEDDLGRGGN

                                               240
              ....|....|....|....|....|....|....|....|....|...
PcSOD3.1      SDSLTTGHAGGRLACGVIGILTE-------------------------
PcSOD3.2      AESGKTGNSGGRIACGVIEVLEE-------------------------
PcSOD1        ELSKTTGNAGGRLACGVIGLAKI-------------------------
HsSOD1        EESTKTGNAGSRLACGVIGIAQ--------------------------
HsSOD3        QASVENGNAGRRLACCVVGVCGPGLWERQAREHSERKKRRRESECKAA
                                                        *
```

Figure S1. Multiple sequence alignment of Cu,Zn-SODs from *P. cochleariae* and *H. sapiens* (SOD1, P00441; SOD3, P08294). Red amino acid residues, metal cation binding and catalysis; yellow residues, cysteine involved in disulfide bridges; green residues, N-glycosylation sites; pink box, signal peptide; torques box, heparin binding region; blue stars, Cu^{2+}-binding; orange stars, Zn^{2+}-binding; black star, position of the *Hs*SOD3 Cys residue involved in the intersubunit disulfide bridge.

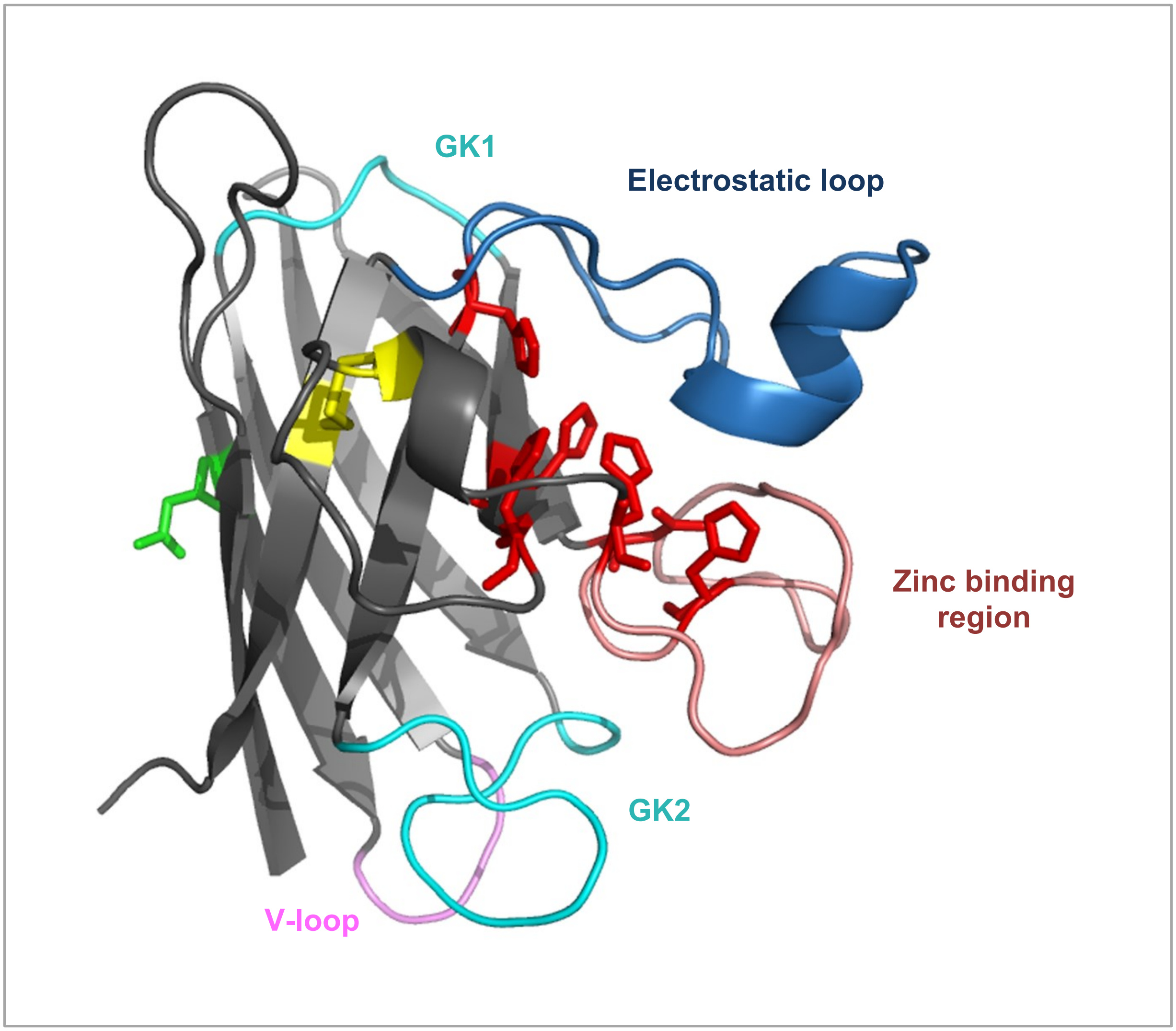

Figure S2. Comparison of the predicted tertiary structure of *Pc*SOD3.1 with the solved HsSOD1 (Uniprot P00441) structure 1HL5 chain A by using Phyre2 (Kelley and Sternberg, 2009). Beta-Strands are displayed as flat arrows, and helices are displayed as ribbons. Amino acid residues ligating metal ions in the active center are shown as red sticks. Key structural elements are color-coded (Perry et al., 2010): dark blue, electrostatic loop (A138-R160) contains charged amino acid residues guiding negatively charged substrates to the active site entrance); light blue, GK1 (G54-G58) and GK2 (D119-I129), Greek key loops 1 and 2; yellow, S-S, disulfide bridge (C74 and C163); purple, V-loop (R42-Q45), variable loop ; light red, Zn^{2+} binding region (H80-D100); green, asparagine residue (N 36) for N-glycosylation.

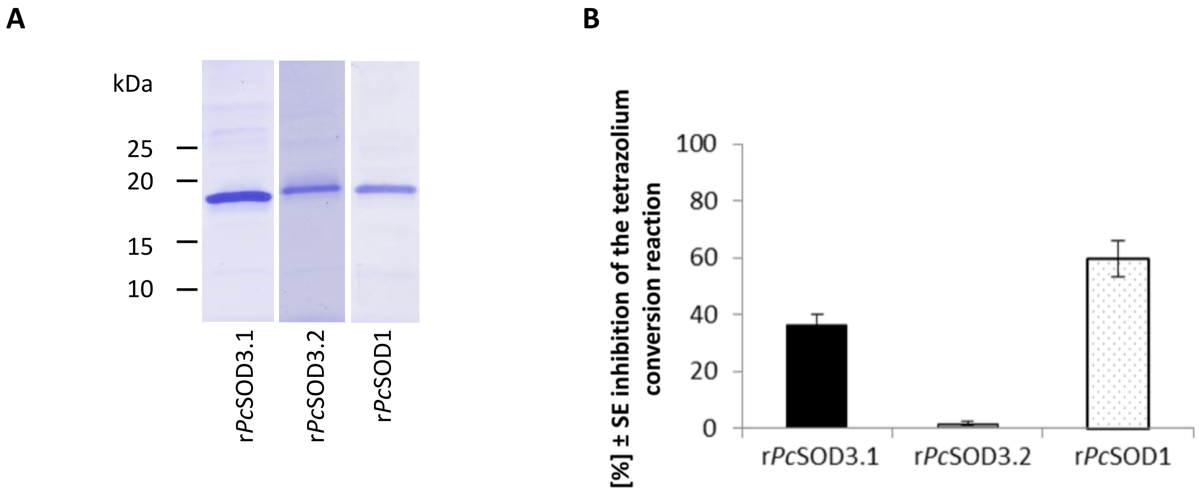

Figure S3. *In vitro* activities of purified recombinant *Pc*SODs. **A**, purified r*PcS*ODs after heterologous expression in *E. coli* cells, left: elution fraction, stained with Coomassie blue. **B**, 4 µg of purified protein each was used for the SOD activity assays. SOD activity was determined colorimetrically at 440 nm as the inhibition of the reduction of the tetrazolium salt, WST-1. (n=3)

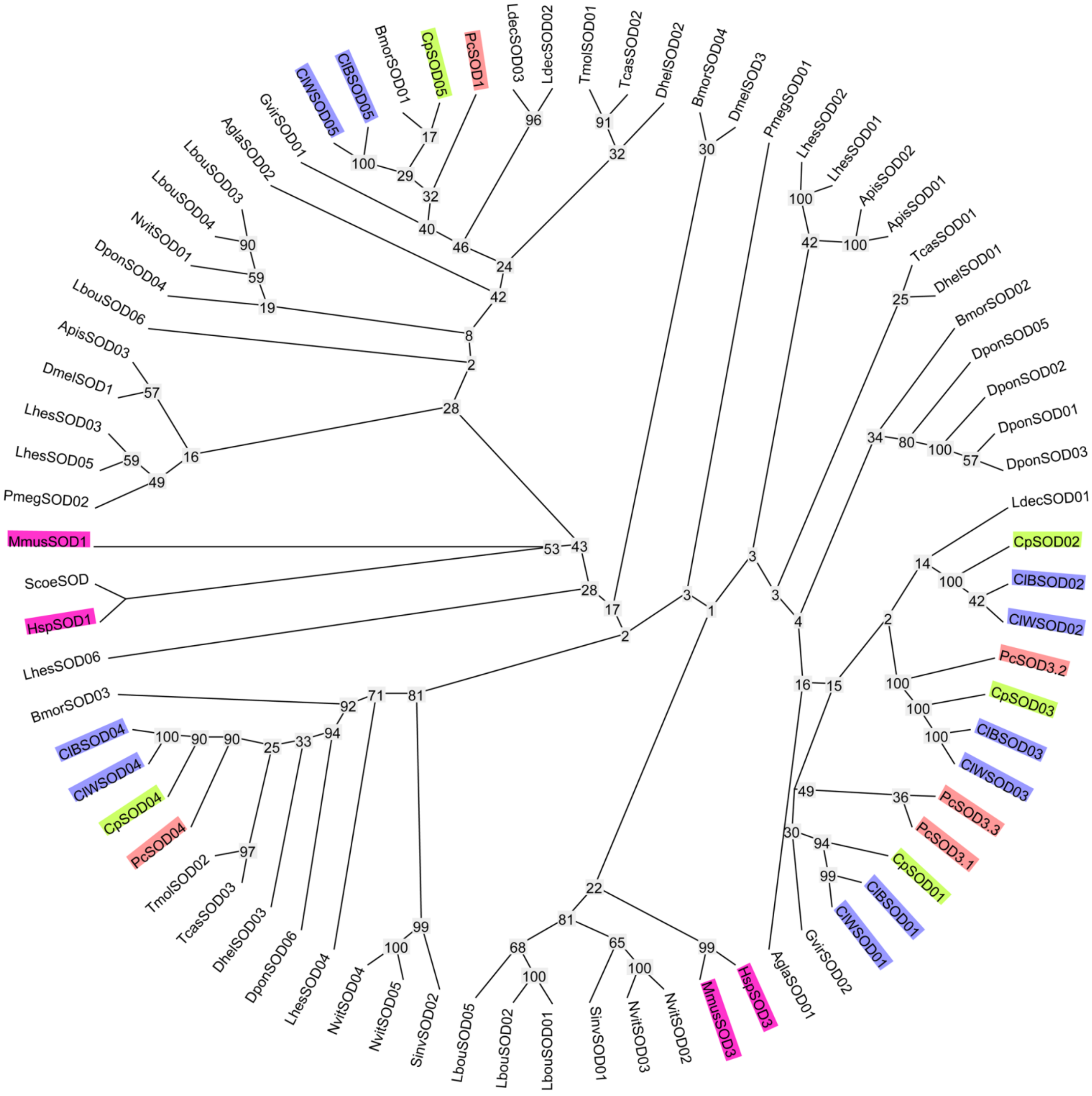

Figure S4A. Phylogenetic tree of SOD1 and SOD3 of selected insect species. Red, *P. cochleariae* (Pc); green, *C. populi* (Cp); blue, *C. lapponica* (ClW, willow-feeder; ClB, birch-feeder); orange, *H. sapiens* (Hsp) and *M. musculus* (Mmus). Numbers at nodes are bootstrap values. Corresponding accession numbers are listed in table S2.

```
                   10        20        30        40        50
          ....|....|....|....|....|....|....|....|....|....|
PcSOD3.1  MFKFAVLAALIT-----ITYAQRSAVVYLFDPSGASGVHGNLTFEQRDSQ
PcSOD3.2  MFKFVVAVVLLSGTYQFCNCDVHSATVRLYDPTGNTEVTGELNFTQVENG
CpSOD03   MFKILVAAAILSVSCHQCNCAIKSAHVKLRDPSGSTEVTGELNFTQTANG
ClSODW03  MFKVLLAAAILSFSCHLCNCAVKTAYVHLRDPSGSTEVTGELNFTQTANG
ClSODB03  MFKVLLAAAILSFSCHLCNCAVKTAYVHLRDPSGSTEVTGELNFTQTANG

                   60        70        80   *    90       100
          ....|....|....|..*.*....|....|....*....|..*.|....|
PcSOD3.1  IQISGEVHGLTPGKHGFHVHQLGNIGLGCLGTGGHFNPHNKHHGAPTDKE
PcSOD3.2  VRITGEIHGIHEGKHGGHVHVDADISKGCIGAGGPFNPHNVRHAGPDDEI
CpSOD03   VRIHGIIHGIHEGLHGGHVHENAKIEASCAGAGGVFNPHKVPHGSPRDEI
ClSODW03  VRIHGKILGIHEGLHGGHVHESANIGRSCAGAGGVFNPHNVPHGSPKDKI
ClSODB03  VRIHGKILGIHEGLHGGHVHESANIGRSCAGAGGVFNPHNVPHGSPKDKI

                  110       120       130       140       150
          .*..*....|....|....|....|....|....|....|..*.|....|
PcSOD3.1  RHVGDLGNIVADATGVAHVHIEDDVIAL-QGNHNIIGRAMVVHAGEDDLG
PcSOD3.2  RNTGDLGNVVGDRFGVAHIDVVDKRIKLNKGIDGIIGRGFVIHEKEDDLG
CpSOD03   RTVGDLGNVIADGSGVAVVDFIDRMVQLNTGVEGVIGRSYVIHEKEDDLG
ClSODW03  RTVGDLGNVVADEYGVAVVDFFDDLVRLNTDVDGVIGRSYVIHEKEDDLG
ClSODB03  RTVGDLGNVVADEYGVAVVDFFDDLVRLNTDVDGVIGRSYVIHEKEDDLG

                  160       170
          ....|....|....|....|....|...
PcSOD3.1  RGGQSDSLTTGHAGGRLACGVIGILTE-
PcSOD3.2  RGNNAESGKTGNSGGRIACGVIEVLEE-
CpSOD03   RGANEESKTTGNCGGLIACGVIEVLEED
ClSODW03  RGNNKESKTNGNCGGLIACGVIEVLEED
ClSODB03  RGNNKESKTNGNCGGLIACGVIEVLEED
```

Figure S4B. Multiple sequence alignment of extracellular Cu,Zn-SODs from Chrysomelina species. Red amino acid residues, metal cation binding and catalysis; blue stars, Cu^{2+}-binding; orange stars, Zn^{2+}-binding.

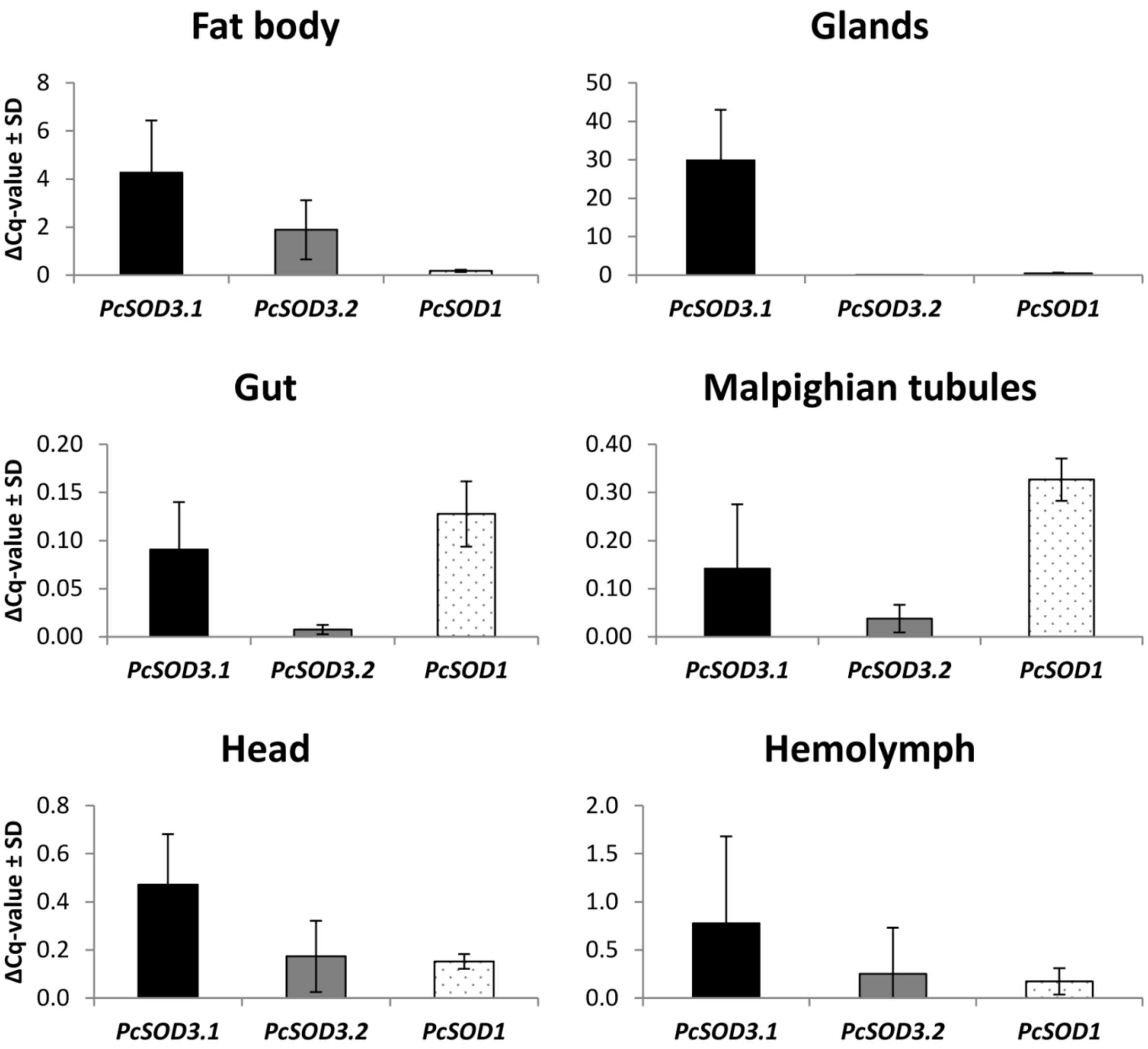

Figure S5. Relative normalized expression levels of *PcSODs* in different tissues of third-instar *P. cochleariae* larvae. Tissues of 3 larvae were pooled for one sample. 4-12 biological replicates were examined. Reference genes were *Pcrps3* and *Pcrpl6*.

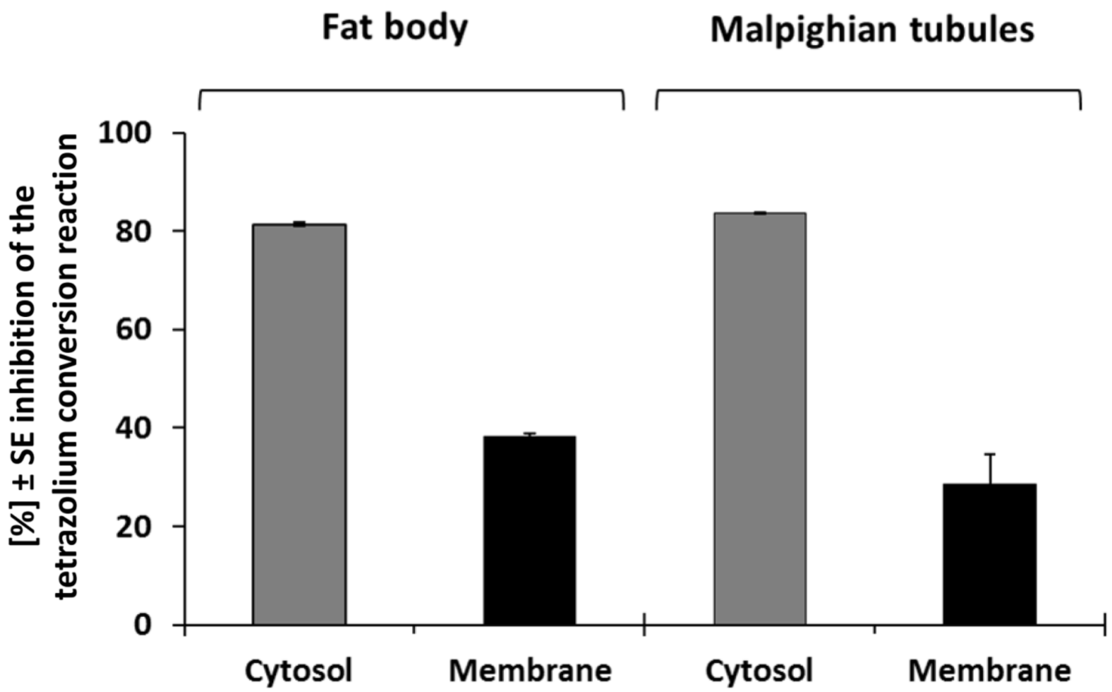

Figure S6. SOD activity after protein isolation. Tissues from six third-instar larvae of *P. cochleariae* were pooled in one sample and proteins were separated into cytosolic and membrane enriched fractions according to the protocol of the Calbiochem ProteoExtract Native Membrane Protein Extraction Kit (Millipore). Protein concentration was measured with bicinchoninic acid protein assay and 6 µg of total protein was used for the SOD activity assay. SOD activity was determined colorimetrically at 440 nm as the inhibition of the reduction of the tetrazolium salt, WST-1. (n=3)

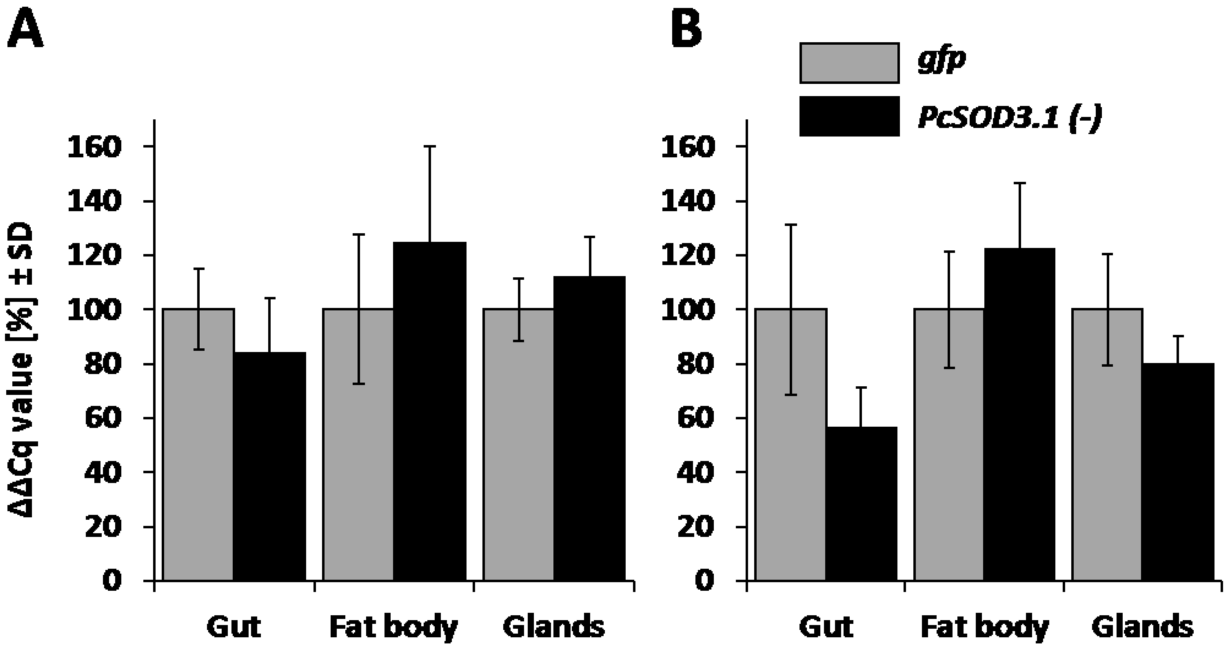

Figure S7. Silencing effects on *PcSOD3.1* expression in different tissues of the larval F1-generation after RNAi induction in the parent generation. A, *P. cochleariae* third-instar larvae were injected with 500 ng of *Pc*SOD3.1-dsRNA or *gfp*-dsRNA and mRNA levels were assayed in larvae of the next generation. B, *P. cochleariae* pupae were injected with 4 µg of *Pc*SOD3.1-dsRNA or *gfp*-dsRNA and mRNA levels were assayed in larvae of the next generation. Reference genes were *PcRPS3* and *PcRPL6.* The ΔCq values of *SOD*-silenced samples have been normalized to the ΔCq values of the *gfp*-injected samples (ΔΔCq). (n=3)

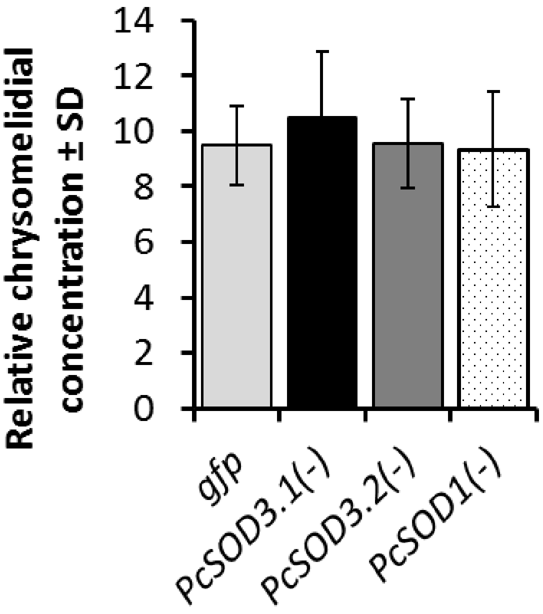

Figure S8. Effect of Cu/Zn SOD silencing on the chrysomelidial synthesis in *P. cochleariae* larvae. GC/MS analysis of secretions was carried out six days after triggering RNAi. The amount of chrysomelidial was normalized to the internal standard methylbenzoate. (n=5)

		PcSOD3.1	PcSOD3.2	PcSOD1
PcSOD3.1(-)	Gut	1,62	189,30	97,29
	Fat body	0,07	108,55	85,82
	MPT	3,30	49,69	98,54
	Glands	0,65	177,77	87,59
PcSOD3.2(-)	Gut	124,12	33,98	56,73
	Fat body	24,75	1,90	81,29
	MPT	96,74	2,38	49,94
	Glands	99,04	19,61	43,98
PcSOD1(-)	Gut	55,31	261,07	6,01
	Fat body	236,28	373,94	6,57
	MPT	43,77	20,81	8,48
	Glands	110,40	65,53	0,44

Figure S9. Expression of Cu/Zn SODs genes after their individual silencing by RNAi in juvenile *P. cochleariae*. 200 ng dsRNA of each Cu/Zn SOD or *gfp* has been used to induce RNAi in early second-instar larvae. RNA from different tissues of *P. cochleariae* larvae has been used as template ten days after ds RNA injection. Reference genes were *PcRPS3* and *PcRPL6.* The ΔCq values of *SOD*-silenced samples have been normalized to the ΔCq values of the *gfp*-injected samples (ΔΔCq). Green: decreased expression level, red: increased expression level. (%means of n=3/treatment)

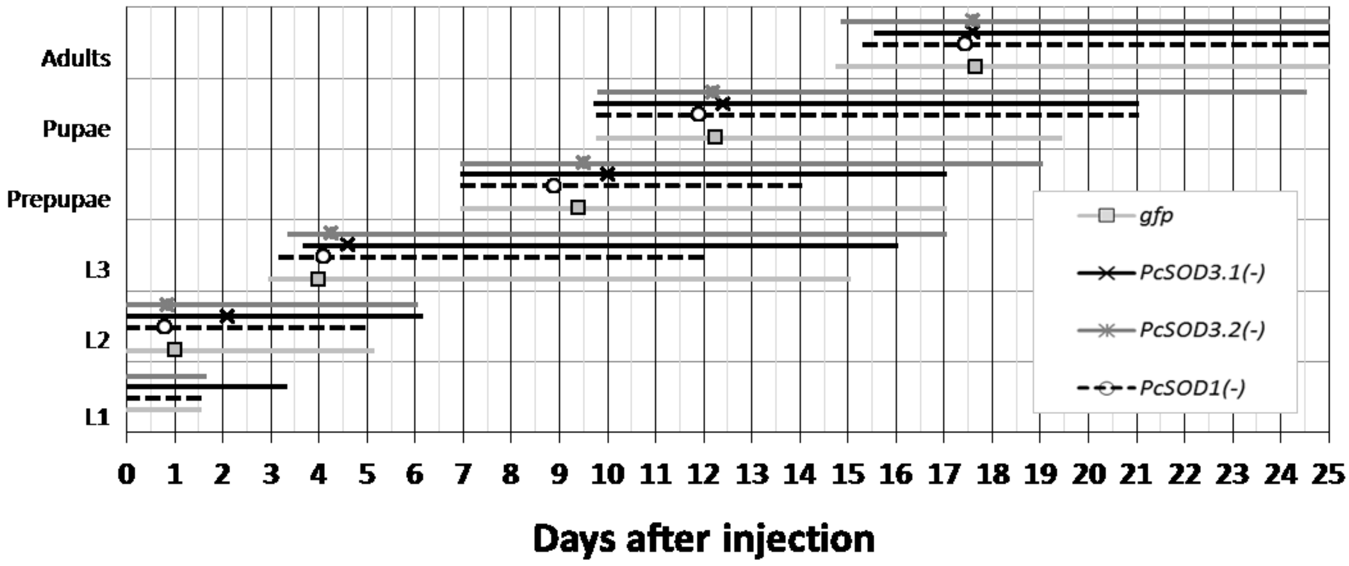

Figure S10. Graphical representation of life stage development of *P. cochleariae*. First-instar larvae were injected with 200 ng of *Pc*SOD3.1-, *Pc*SOD3.2-, *Pc*SOD1-dsRNA or *gfp*-dsRNA. Markers indicate 50% of population in this state, lines represent first and last turnover. L1-L3, larval instars.

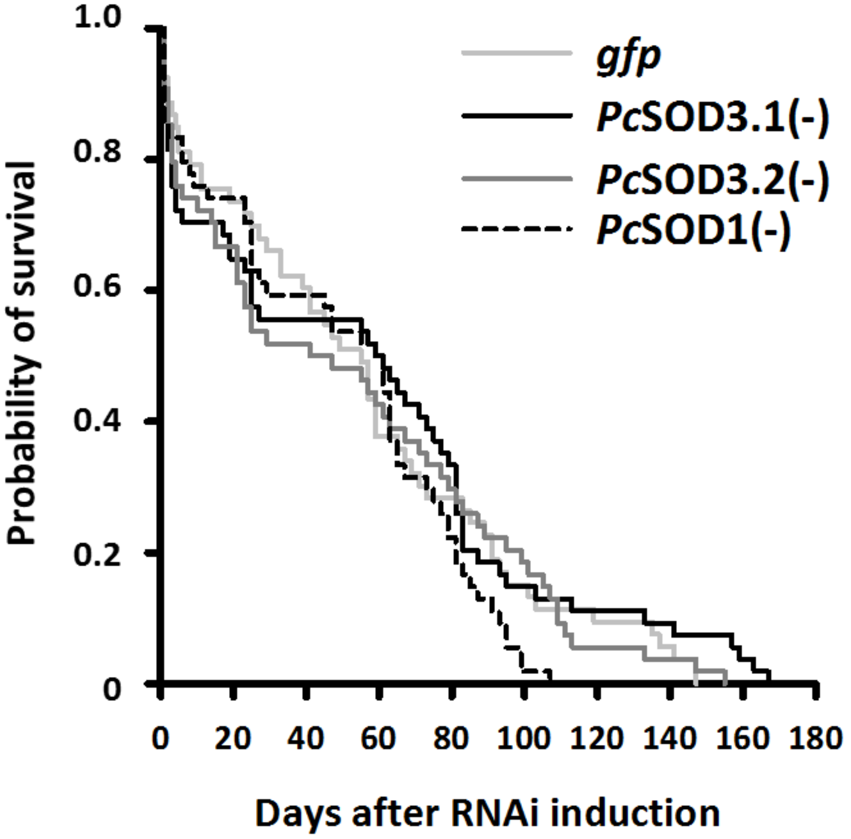

Figure S11. Longevity of *P. cochleariae* after silencing of Cu/Zn SODs by RNAi. The probability of survival is depicted as Kaplan-Meier curves by applying Gehan-Breslow statistic test. Second-instar larvae were injected with 200 ng of *PcSOD3.1*-, *PcSOD3.2-, PcSOD1*-dsRNA or *gfp*-dsRNA (n=60/treatment).

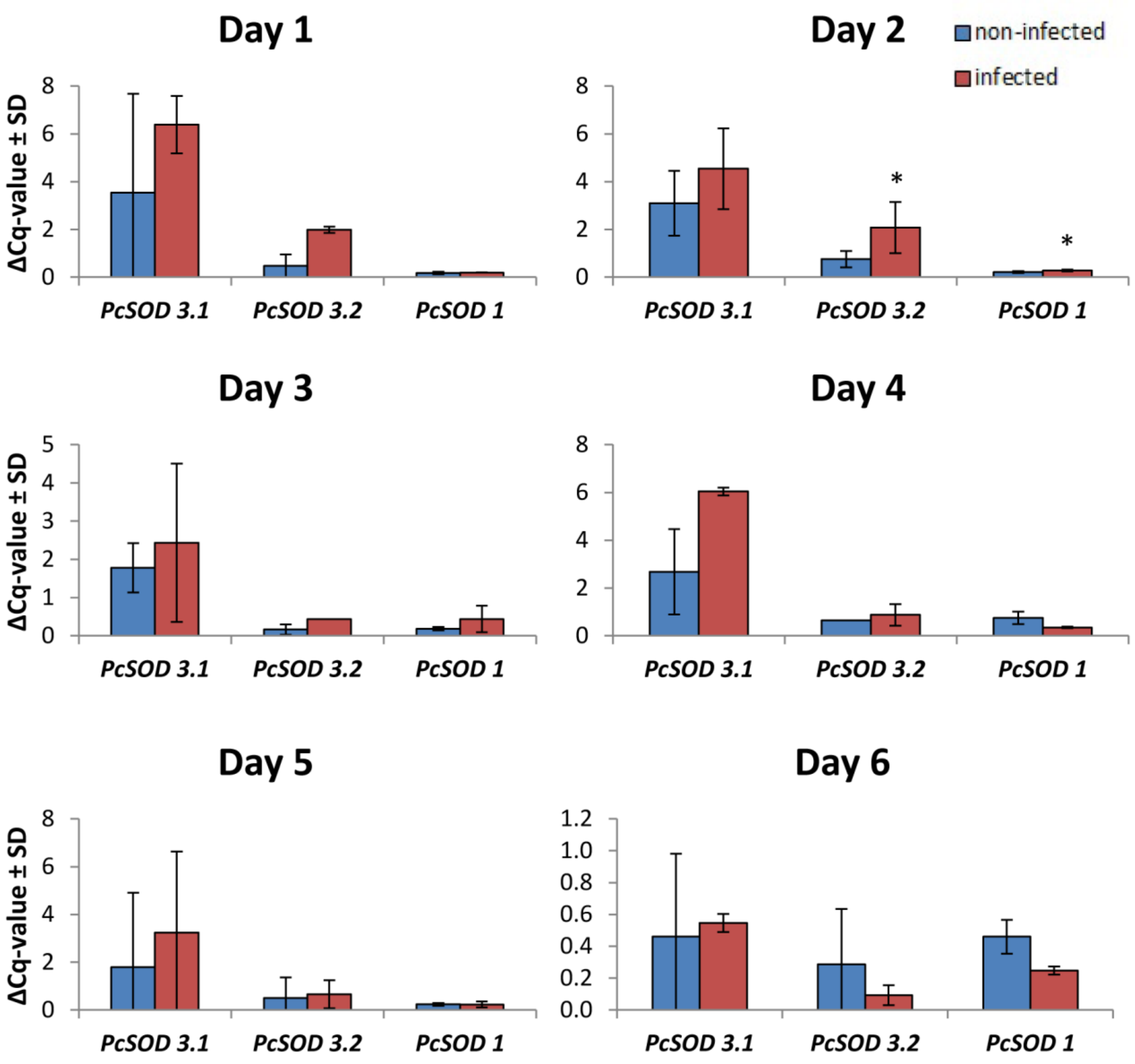

Figure S12. Relative normalized expression levels of *sod*'s in whole third-instar *P. cochleariae* larvae 1 to 6 days after infection with *M. anisopliae*. Non-infected and infected larvae in comparison, 1 to 6 biological replicates of whole larvae were examined. Reference genes were *PcRPS3* and *PcRPL6*. * = significantly different to their non-infected group ($p<0.05$, two-tailed t-test).

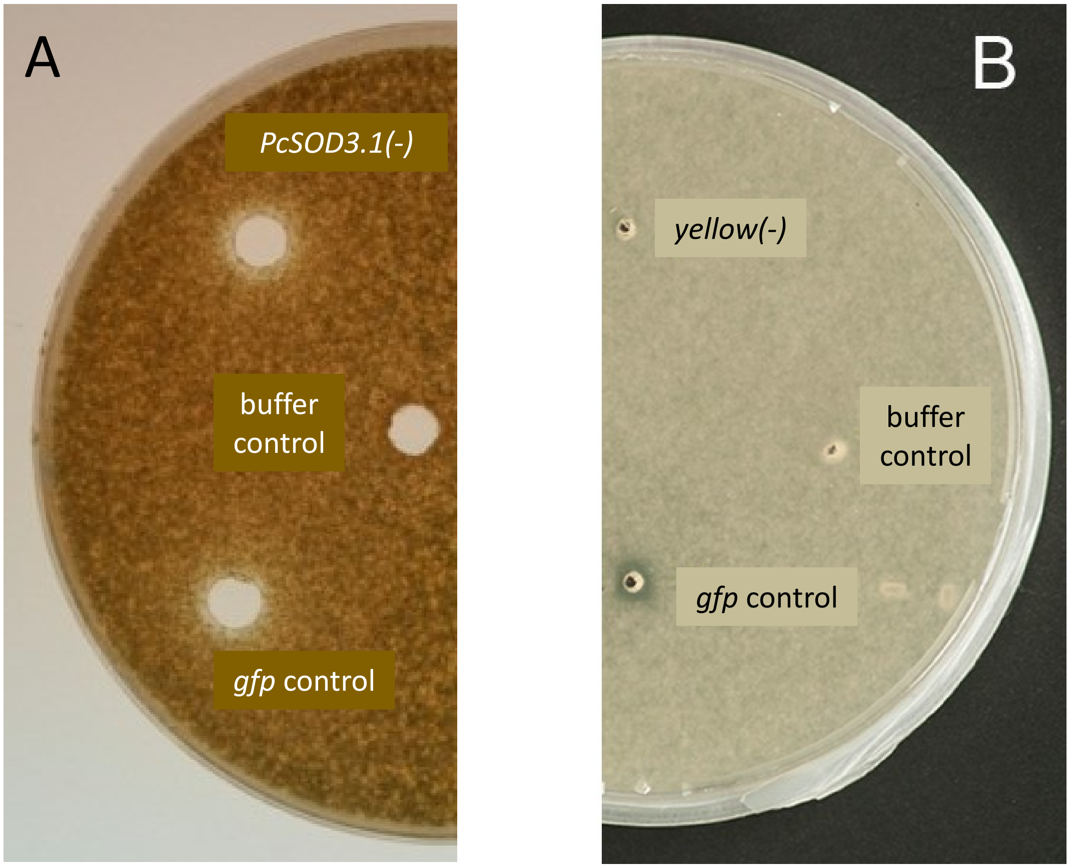

Figure S13. Effect of defensive secretions from juvenile *P. cochleariae* on the growth of *Metarhizium anisopliae* in agar diffusion tests after three days of incubation. A, Effect of secretions collected from *PcSOD3.1* silenced larvae (1:10 (w/v) in 50 mM $NaPO_4$ pH 5.8, 10 µl per spot); B, Effect of secretions collected from *yellow* silenced larvae. (1:30 (w/v) in 50 mM $NaPO_4$ pH 5.8, 5 µl per spot).

Kelley, L.A., Sternberg, M.J.E., 2009. Protein structure prediction on the Web: a case study using the Phyre server. Nat. Protoc. 4, 363-371.
Perry, J.J.P., Shin, D.S., Getzoff, E.D., Tainer, J.A., 2010. The structural biochemistry of the superoxide dismutases. Biochim. Biophys. Acta 1804, 245-262.

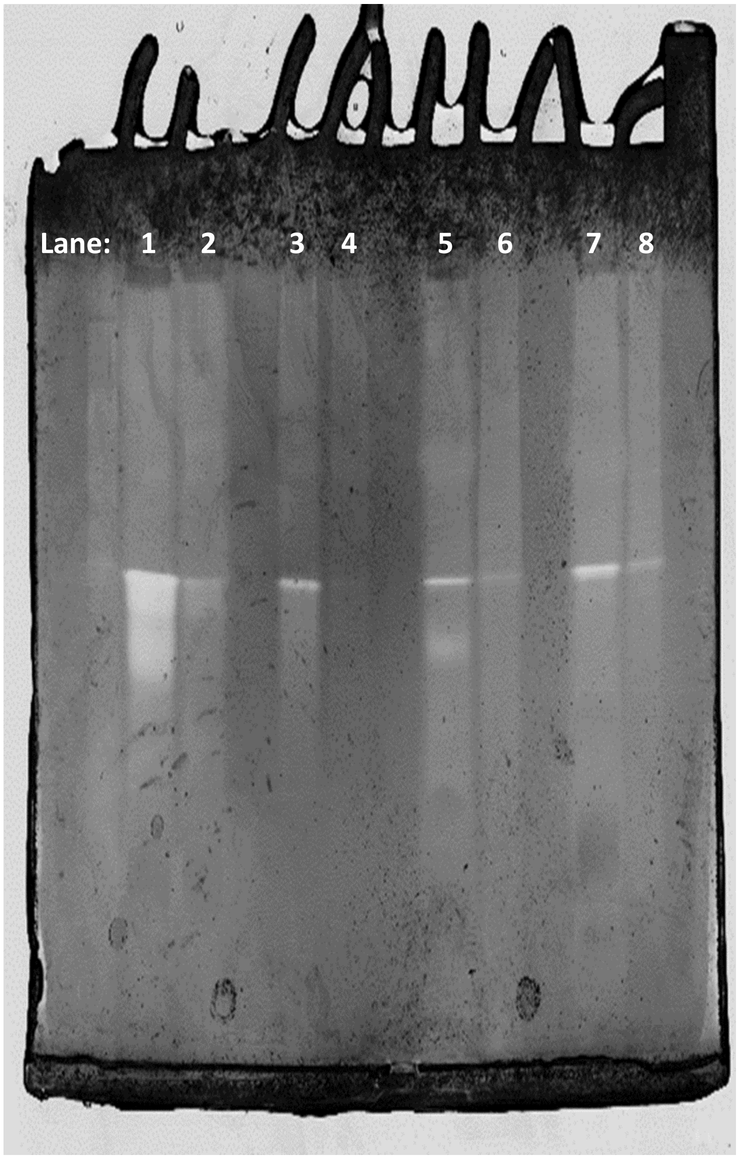

Figure S14. Full-length gel corresponds to figure 1A in the main text. It shows the detection of SOD activity in defensive exudates and hemolymph collected from larvae of different leaf beetle species. From each species 1 µl hemolymph and 0.3 µl secretion was separated on native PAGE and functionally stained for SOD activity by using a qualitative zymography method based on the conversion of the tetrazolium salt, MTT. SOD activity inhibits the formation of a dark blue formazane dye and corresponds to white bands on a dark background.

Lane 1/2, *P. cochleariae*, hemolymph/secretion; lane 3/4, bovine erythrocyte SOD; lane 5/6, *Gastrophysa viridula*, hemolymph/ secretion; lane 7/8, *Chrysomela populi*, hemolymph/ secretion.

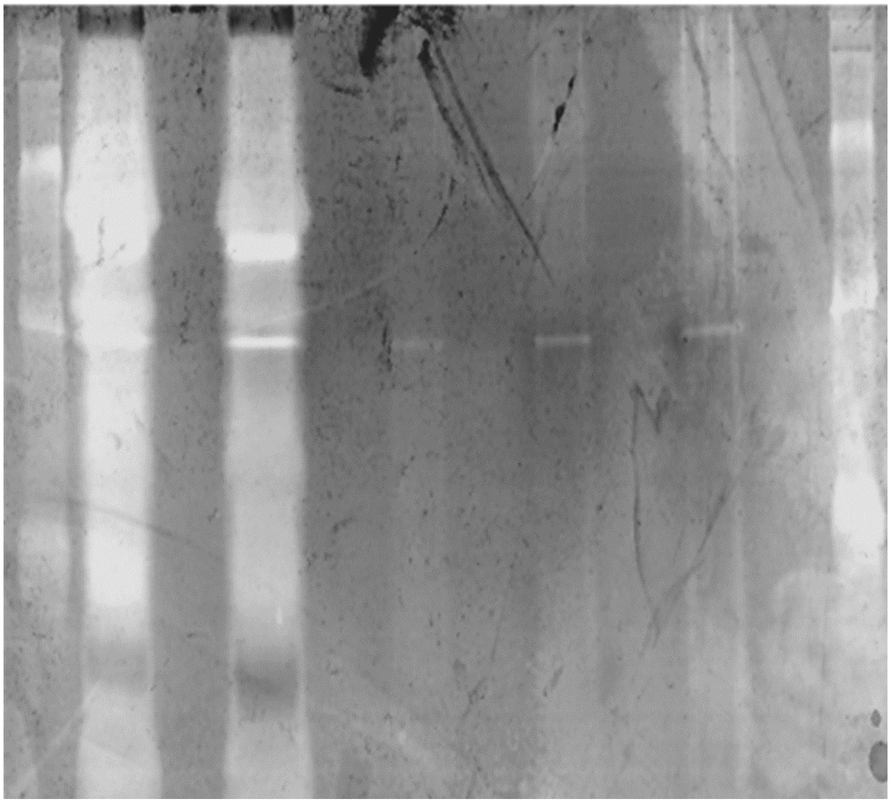

Figure S15. Full-length gel corresponds to figure 1A in the main text. It shows the detection of SOD activity in defensive exudates and hemolymph collected from larvae of *Chrysomela lapponica* (C. lapp.) One µl hemolymph and 0.3 µl secretion was separated on native PAGE and functionally stained for SOD activity by using a qualitative zymography method based on the conversion of the tetrazolium salt, MTT. SOD activity inhibits the formation of a dark blue formazane dye and corresponds to white bands on a dark background.

Lane 1, C. lapp. affiliated to *Salix* ssp., hemolymph; lane 2, C. lapp. affiliated to *Betula* ssp., hemolymph; lane 3, C. lapp. affiliated to *Salix* ssp., secretion; lane 4, C. lapp. affiliated to *Betula* ssp., secretion.

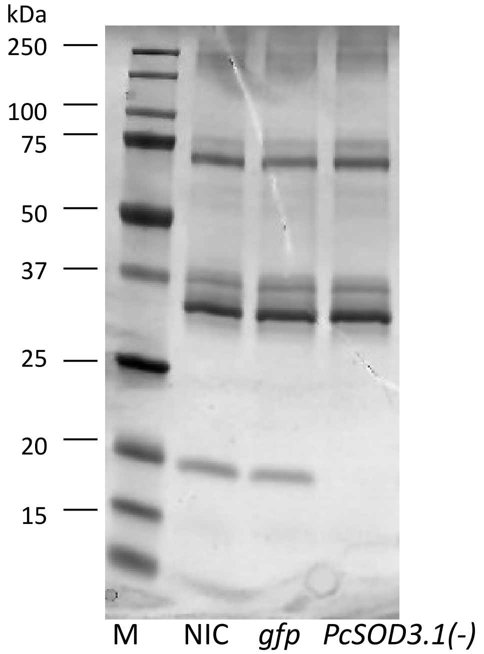

Figure S16. Full-length gel corresponds to figure 2C in the main text. Coomassie stained SDS-PAGE with 0.3 µl of larval defence secretions from *P. cochleariae* nine days after RNAi induction. Injection was carried out with 200 ng *PcSOD3.1*-dsRNA or 200 ng *gfp* as a control. M, marker (Bio-Rad Laboratories, Munich, Germany); NIC, non-injection control.

SUPPLEMENTARY TABLES

Table S1. Peptides identified from superoxide dismutases from juvenile *P. cochleariae*.

Peptides identified from *Pc*SOD 3.1 in hemolymoph.

peptide sequence[a]	theor. $[M+H]^{+}$ [b]	exp. $[M+H]^{+}$ [c]	mass error (ppm)	peptide score	retention time (min)
DSQIQISGEVHGLTPGK	1765.9177	1765.9156	4.4	8.0	33.07
HVGDLGNIVADATGVAHVHIEDDVIALQGNHNIIGR	3739.9205	3739.9205	-1.6	7.8	43.86
AMVVHAGEDDLGR	1369.6487	1369.6507	-1.6	7.7	26.37
GGQSDSLTTGHAGGR	1400.6741	1400.6707	13.9	6.3	25.34
AM_{ox}VVHAGEDDLGR	1385.6495	1385.6515	2.7	7.9	23.16

→ exemplary table of identified peptides from *Pc*SOD3.1 in hemolymph; results for defensive secretions are similar

Peptides identified from *Pc*SOD 3.2 in hemolymoph.

peptide sequence[a]	theor. $[M+H]^{+}$ [b]	exp. $[M+H]^{+}$ [c]	mass error (ppm)	peptide score	retention time (min)
NTGDLGNVVGDR	1216.6014	1216.5986	5.7	7.9	28.06
FGVAHIDVVDKR	1355.7344	1355.7367	-4.7	7.7	30.36
ITGEIHGIHEGK	1290.6637	1290.6663	-10.7	7.6	21.63
GIDGIIGR	800.4588	800.46	-3.1	7.5	31.62
GC*IGAGGPFNPHNVR	1552.7408	1552.7418	-1.3	7.3	29.52
GNNAESGKTGNSGGR	1405.6543	1405.6495	5.7	6.1	29.74

Peptides identified from *Pc*SOD1 in Malpighian tubules and fat body.

peptide sequence[a]	theor. $[M+H]^{+}$ [b]	exp. $[M+H]^{+}$ [c]	mass error (ppm)	peptide score	retention time (min)
TVVVHADPDDLGQGGHELSK	2074.0231	2074.02	1.5	6.4	28.96
FISLEGENSIIGR	1434.7636	1434.7587	3.4	6.2	40.59
LAC*GVIGLAK	1001.5863	1001.5813	4.9	6.2	33.78
GTVFFNQANSTSPVEVTGEITGLQK	2624.3185	2624.3202	-0.7	5.6	42.78
HIGDLGNIQATDNGVAK	1722.893	1722.8769	9.3	5.4	43.38
IYC*IVELGTESFIIGNYNK	2233.118	2233.121	-1.3	5.7	40.36

→ exemplary table of identified peptides from *Pc*SOD1 in Malpighian tubules; results for fat body are similar

[a] C*, C-carbamidomethylation; Mox, M-oxidation

[b] theoretical monoisotopic mass of the peptide

[c] calculated monoisotopic mass of the lock-mass corrected peptide ion cluster

Table S2. Low molecular proteins identified in hemolymph from juvenile *P. cochleariae.*

name	similar to protein	fmol protein			
		NIC	*E. coli*	*M. luteus*	*M. anisopliae*
PcAMP01	acaloleptin A (coleoptericin)	252.93	3354.64	884.15	1750.57
PcAMP02	attacin-like immune protein (attacin_C)	27.89	1536.42	1419.46	634.22
PcAMP03	defensin_2	0	659.06	309.20	123.43

Peptides identified from PcAMP01

peptide sequence[a]	theor. $[M+H]^{+\,b}$	exp. $[M+H]^{+\,c}$	mass error (ppm)	peptide score	retention time (min)
QQPWEVSPNINR	1467.7278	1467.7339	-4.2	8.9	35.13
SLQPGAPDINNGK	1310.6626	1310.6699	-5.6	7.7	25.51
AKPTWHAGGTIR	1294.7073	1294.7015	4.5	8.6	21.07
SLQPGAPDINNDKK	1496.7773	1496.7703	4.6	8.0	23.55
QQPWEVSPNINRDGAGNVR	2137.0648	2137.0533	5.3	8.1	33.49
DHDFNAGWGK	1146.4921	1146.4963	-3.7	7.5	27.08
SLQPGAPDINNDK	1368.6796	1368.6754	3.1	7.7	26.40
TDIEVQNHGKDHDFNAGWGK	2268.0656	2268.0428	10.0	6.7	33.71
AKPTWHAGGTIR	1294.701	1294.7015	-0.4	7.9	81.18
TDIEVQNHGK	1140.5675	1140.5644	2.7	7.5	17.31
SLQPGAPDINNGKK	1438.7498	1438.7649	-10.5	8.1	23.33
TDIEVQNHGKDHDFNAGWGK	2268.1397	2268.0428	42.7	7.2	41.40
DHDFNAGWGK	1146.5051	1146.4963	7.7	7.4	80.92
VSPNINRDGAGNVR	1468.7331	1468.7615	-19.3	6.6	45.14
KQQPWEVSPNINR	1595.8306	1595.8289	1.1	8.5	31.31

Peptides identified from PcAMP02

peptide sequence[a]	theor. $[M+H]^{+\,b}$	exp. $[M+H]^{+\,c}$	mass error (ppm)	peptide score	retention time (min)
LDYTHTPSGSGAFIQADR	1935.9195	1935.9259	3.3	9.0	32.93
YNIYTSPK	985.4989	985.4993	0.4	8.4	25.63
KDFGVDATAQYQR	1498.7285	1498.7335	3.4	8.6	26.76
NYGTDVAAGGK	1052.5007	1052.5005	-0.2	8.2	20.54
DFGVDATAQYQR	1370.6335	1370.6495	11.7	8.5	32.01
GGRLDYTHTPSGSGAFIQADR	2206.0636	2206.0589	-2.1	7.3	30.70
TRNYGTDVAAGGK	1309.6495	1309.6444	-3.9	7.4	80.87

Peptides identified from PcAMP03

peptide sequence[a]	theor. [M+H]+ [b]	exp. [M+H]+ [c]	mass error (ppm)	peptide score	retention time (min)
VTCDVLNFEIK	1337.677	1337.679	1.5	6.4	42.09
LNDAACAAHCLTLGK	1614.7727	1614.7946	13.5	5.2	37.38

[a] C*, C-carbamidomethylation; Mox, M-oxidation

[b] theoretical monoisotopic mass of the peptide

[c] calculated monoisotopic mass of the lock-mass corrected peptide ion cluster

Table S3. Proteome analysis via shotgun from hemolymph of *P. chochleariae* larvae

protein	fmol protein				
	gfp not infected	*gfp* infected with *M. anisopliae*	*PcSOD3.1*(-) not infected	*PcSOD3.1*(-) infected with *M. anisopliae*	*PcSOD3.2* (-) not infected
*Pc*SOD3.1	5.48 ± 3.67	3.76 ± 4.43	0	0	1.38 ± 2.75
*Pc*SOD3.2	14.49 ± 1.11	9.07 ± 3.33	16.25 ± 8.18	8.77 ± 3.8	0

Table S4. Peptides identified from PcYellow-like in secretions of juvenile *P. cochleariae*.

peptide sequence[a]	theor. [M+H]+ [b]	exp. [M+H]+ [c]	mass error (ppm)	peptide score	retention time (min)
NNPFAAPQIFHTYR	1675.834	1675.841	4.4	8.6	40.78
IHSGTPVTLGTVTDR	1553.828	1553.831	1.8	8.7	33.12
QANIDGGYFR	1140.543	1140.546	2.1	8.4	33.59
TNHLLLR	866.5207	866.5207	0.0	8.6	30.37
LLAFDLR	847.5036	847.5021	-1.8	8.0	42.40
QELWILTSR	1145.631	1145.633	1.8	8.3	43.78
NTFVYLGDVR	1183.611	1183.618	5.9	8.0	39.71
ITIDECER	1035.478	1035.483	4.8	6.7	28.24
STQSSAEAIDR	1164.549	1164.552	2.1	8.0	23.61
SWAVR	618.3358	618.3402	7.1	7.2	28.49
DLSIVHEWSQIEFDYHSEVER	2618.216	2618.219	1.0	7.8	47.43
LWVIDIGPLNDVITCPAR	2052.095	2052.1	2.4	8.0	53.14
STQSSAEAIDRDGIMYYGLLGDIK	2603.266	2603.338	27.9	6.0	41.85
RDLSIVHEWSQIEFDYHSEVER	2774.317	2774.334	6.3	6.0	45.35
GRQELWILTSR	1358.754	1358.764	7.7	8.2	40.53
RLFYHAMSSPTENWVYTSYLR	2621.261	2621.228	-12.3	6.3	30.43
LFYHAMSSPTENWVYTSYLR	2481.154	2481.164	3.7	8.1	44.43

AQYVASGTMFDVNETNFR	2065.928	2065.933	2.1	8.1	37.70

[a] C*, C-carbamidomethylation; Mox, M-oxidation

[b] theoretical monoisotopic mass of the peptide

[c] calculated monoisotopic mass of the lock-mass corrected peptide ion cluster

Table S5. Primer used for cloning, quantitative real time PCR, dsRNA production.

Primer type	Target	↔	Sequence	Amplicon length
Coding sequence	*PcSOD1*	→	ATGCCTACAAAAGCAGTTTGCGTTCTC	462
		←	CTATATTTTTGCCAATCCAATGACTCCA	
	PcSOD3.1	→	GAGAAAAAAATTGATACTTCG	670
		←	TTTTCACTTATCTAAAATCGTTTAT	
	PcSOD3.2	→	ATGTTCAAGTTTGTAGTAGCCGTGGTTT	553
		←	CTCCCTAATCGTAGGTTATTCATTCTTCC	
dsRNA template	*PcSOD1*	→	TAATACGACTCACTATAGGGAGACTCCAATGTGGCGAATA	243
		←	TAATACGACTCACTATAGGGAGATGCCTACAAAAGCAGTT	
	PcSOD3.1	→	TAATACGACTCACTATAGGGAGAACATCGTAGCCGACGCA	193
		←	TAATACGACTCACTATAGGGAGACCGATGACCCCGCAA	
	PcSOD3.2	→	TAATACGACTCACTATAGGGAGAGACTCCGCAAGCTATAC	508
		←	TAATACGACTCACTATAGGGAGACAAGTTTGTAGTAGCCG	
	gfp	→		523
		←	TAATACGACTCACTATAGGGAGATAATCCCAGCAGCAGTT	
	PcYellow-like	→	GTAATACGACTCACTATAGGGAGCTTTCTTGGCGTCACTGGTGGTAG	
		←	GTAATACGACTCACTATAGGGAGCCAGCAAACCATAATACATTATTCCATCC	
qPCR	*PcSOD1*	→	ATCATGATCTGGGCCACCATGC	77
		←	ACAACACCAACGGGTGCATTTCC	
	PcSOD3.1	→	AAACATCGTAGCCGACGCAACAG	89
		←	TGCCGATGATGTTGTGGTTGC	
	PcSOD3.2	→	TCAATGTGAGCGACGCCGAAAC	111
		←	TGGACCTTTCAATCCCCACAACG	
	PcRP-L6	→	CAAGACAGGATCAGACCTAGAC	126
		←	CACTCTCTTTCCTTTGTGGGAAC	
	PcRP-S3	→	AAGGCTGTGAAGTTGTGGTATCTGG	128
		←	ATGGCGGGTGGCAGTGTCTAC	
pET100 Insert	*PcSOD1*	→	CACCATGCCTACAAAAGCAGTTTGC	466
		←	CTATATTTTTGCCAATCCAATGACT	

	PcSOD3.1	→	CACCATGCGTAGCGCAGTCGTCTATCT	487
		←	AGCCAGAACGCGTATTTACTC	
	PcSOD3.2	→	CACCGATGTGCATTCAGCAACAGTT	478
		←	TCATTCTTCCAATACTTCGA	

Table S6. Accession numbers of sequences used for all the studies of this publication.

Species	Designation in figure	Accession number
Anoplophora glabripennis	AglaSOD01	gi|550258789|gb|JAB66410.1|
	AglaSOD02	gi|550254905|gb|JAB65445.1|
Acyrthosiphon pisum	ApisSOD01	gi|242247393|ref|NP_001156153.1|
	ApisSOD02	gi|239789313|dbj|BAH71287.1|
	ApisSOD03	gi|242247211|ref|NP_001156243.1|
Bombyx mori	BmorSOD01	gi|112982998|ref|NP_001037084.1|
	BmorSOD02	gi|512930962|ref|XP_004932190.1|
	BmorSOD03	gi|512885538|ref|XP_004921611.1|
	BmorSOD04	gi|68144076|gb|AAY86076.1|
Chrysomela lapponica	ClBSOD01	gb|KT002136
	ClBSOD02	gb|KT002134
	ClBSOD03	gb|KT002135
	ClBSOD04	gb|KT002137
	ClBSOD05	gb|KT002138
	ClWSOD01	gb|KT002139
	ClWSOD02	gb|KT002140
	ClWSOD03	gb|KT002141
	ClWSOD04	gb|KT002142
	ClWSOD05	gb|KT002143
Chrysomela populi	CpSOD01	gb|KT270576
	CpSOD02	gb|KT270577
	CpSOD03	gb|KT270578
	CpSOD04	gb|KT270579
	CpSOD05	gb|KT270580
Phaedon cochleariae	PcSOD1	gb|KT002146
	PcSOD3.1	gb|JQ424878
	PcSOD3.2	gb|KT002145
	PcSOD3.3	gb|KT002144
	PcSOD04	gb|KT270585
	PcAMP01	gb|KT270583
	PcAMP02	gb|KT270582
	PcAMP03	gb|KT270581
	PcYellow-like	gb|KT270584
	PcRPL6	gb|KT270586

	PcRPS3	gb|KC109783
Dastarcus helophoroides	DhelSOD01	gi|667755291|gb|AIG92782.1|
	DhelSOD02	gi|667755289|gb|AIG92781.1|
	DhelSOD03	gi|667755293|gb|AIG92783.1|
Drosophila melanogaster	DmelSOD1	|Q7JR71|Q7JR71_DROME
	DmelSOD3	gi|17136496|ref|NP_476735.1|
Dendroctonus ponderosae	DponSOD01	gi|546674530|gb|ERL85890.1|
	DponSOD02	gi|478251452|gb|ENN71916.1|
	DponSOD03	gi|332380651|gb|AEE65524.1|
	DponSOD04	gi|332376316|gb|AEE63298.1|
	DponSOD05	gi|332376945|gb|AEE63612.1|
	DponSOD06	gi|478253114|gb|ENN73487.1|
Homo sapiens	HspSOD1	|P00441|SODC_HUMAN
	HspSOD3	|P08294|SODE_HUMAN
Leptopilina boulardi	LbouSOD01	gi|357535425|gb|AET83767.1|
	LbouSOD02	gi|357535429|gb|AET83769.1|
	LbouSOD03	gi|357535423|gb|AET83766.1|
	LbouSOD04	gi|357535431|gb|AET83770.1|
	LbouSOD05	gi|357535433|gb|AET83771.1|
	LbouSOD06	gi|506969309|gb|AGM32998.1|
Lygus hesperus	LhesSOD01	gi|732607915|gb|JAG19381.1|
	LhesSOD02	gi|732607911|gb|JAG19379.1|
	LhesSOD03	gi|732613469|gb|JAG22158.1|
	LhesSOD04	gi|732610069|gb|JAG20458.1|
	LhesSOD05	gi|732576017|gb|JAG03432.1|
	LhesSOD06	gi|732622201|gb|JAG26524.1|
Mus musculus	MmusSOD1	sp|P08228|SODC_MOUSE
	MmusSOD3	sp|O09164|SODE_MOUSE
Nasonia vitripennis	NvitSOD01	gi|156548615|ref|XP_001608103.1|
	NvitSOD02	gi|345481696|ref|XP_001602916.2|
	NvitSOD03	gi|645035824|ref|XP_008216838.1|
	NvitSOD04	gi|645035835|ref|XP_008216841.1|
	NvitSOD05	gi|645035832|ref|XP_008216840.1|
Panstrongylus megistus	PmegSOD01	gi|656761599|gb|JAC85673.1|
	PmegSOD02	gi|656761385|gb|JAC85566.1|
Solenopsis invicta	SinvSOD01	gi|322782495|gb|EFZ10444.1|
	SinvSOD02	gi|322782511|gb|EFZ10460.1|

Tribolium castaneum	TcasSOD01	|D6X0S2|D6X0S2_TRICA
	TcasSOD02	|D2A2T2|D2A2T2_TRICA
	TcasSOD03	gi|91091194|ref|XP_972244.1|
Tenebrio molitor	TmolSOD01	gi|669688118|gb|AII26027.1|
	TmolSOD02	gi|669688116|gb|AII26026.1|
Streptomyces coelicolor	ScoeSOD	|O51917|SODF_STRCO
